# Fast NMR method to probe solvent accessibility and disordered regions in proteins

**DOI:** 10.1038/s41598-018-37599-z

**Published:** 2019-02-07

**Authors:** André F. Faustino, Glauce M. Barbosa, Micael Silva, Miguel A. R. B. Castanho, Andrea T. Da Poian, Eurico J. Cabrita, Nuno C. Santos, Fabio C. L. Almeida, Ivo C. Martins

**Affiliations:** 1 Instituto de Medicina Molecular, Faculdade de Medicina, Universidade de Lisboa, Av. Prof. Egas Moniz, 1649-028 Lisbon, Portugal; 2 Instituto de Bioquímica Médica Leopoldo de Meis, Universidade Federal do Rio de Janeiro, Rio de Janeiro, 21941-902 RJ Brazil; 3 Centro Nacional de Ressonância Magnética Nuclear, Universidade Federal do Rio de Janeiro and National Institute of Structural Biology and Bioimage (CENABIO), Rio de Janeiro, 21941-902 RJ Brazil; 4 UCIBIO, Departamento de Química, Faculdade de Ciências e Tecnologia, Universidade Nova de Lisboa, Quinta da Torre, 2829-516 Monte de Caparica Portugal; 5 Present Address: iBET, Instituto de Biologia Experimental e Tecnológica, Apartado 12, 2780-901 Oeiras, Portugal

## Abstract

Understanding protein structure and dynamics, which govern key cellular processes, is crucial for basic and applied research. Intrinsically disordered protein (IDP) regions display multifunctionality via alternative transient conformations, being key players in disease mechanisms. IDP regions are abundant, namely in small viruses, allowing a large number of functions out of a small proteome. The relation between protein function and structure is thus now seen from a different perspective: as IDP regions enable transient structural arrangements, each conformer can play different roles within the cell. However, as IDP regions are hard and time-consuming to study via classical techniques (optimized for globular proteins with unique conformations), new methods are required. Here, employing the dengue virus (DENV) capsid (C) protein and the immunoglobulin-binding domain of streptococcal protein G, we describe a straightforward NMR method to differentiate the solvent accessibility of single amino acid N-H groups in structured and IDP regions. We also gain insights into DENV C flexible fold region biological activity. The method, based on minimal pH changes, uses the well-established ^1^H-^15^N HSQC pulse sequence and is easily implementable in current protein NMR routines. The data generated are simple to interpret, with this rapid approach being an useful first-choice IDPs characterization method.

## Introduction

Nuclear magnetic resonance (NMR) spectroscopy is the technique of excellence to obtain structural and dynamics atomic resolution information of macromolecules, especially proteins^1^. NMR is compatible with room temperature solution measurements, a major advantage over other high-resolution structural techniques (X-ray crystallography and cryo-electron microscopy). Via NMR, protein structures can now be determined with chemical shifts data alone (employing the CS-Rosetta package)^2–6^, which is extremely important when merely sparse data is available^3,4^. Noteworthy, NMR provides protein dynamics information in physiological conditions, via the probing of different macromolecular motion timescales^7–9^. Backbone amide hydrogen exchange experiments are particularly informative of protein N-H solvent accessibility, being related to both structure and dynamics (sensitive to the millisecond timescale)^9–13^. Nevertheless, complementary data is frequently still necessary^14–18^, especially to study intrinsically disordered protein (IDP) regions^17,19^. Therefore, there is a major unmet demand for fast straightforward methodologies to analyze IDP regions, which play key roles in health and disease mechanisms.

**Figure 1:**
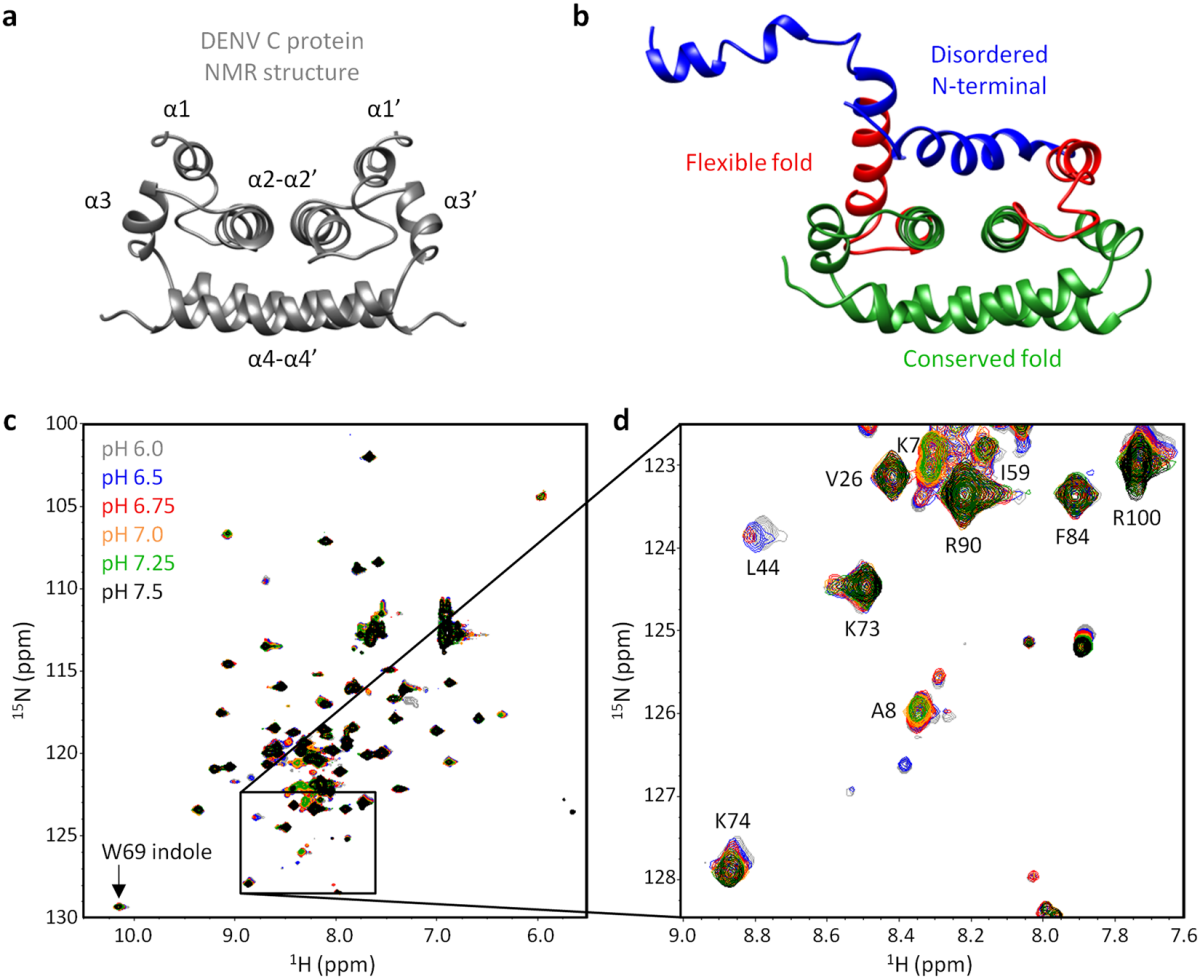
DENV C structure, overall dynamics and ^1^H-^15^N HSQC spectra from pH 6.0 to 7.5. (**a**) DENV C homodimer experimental structure. This protein is positively charged, with 26 cationic and 2 anionic out of 100 residues *per* monomer. From amino acid residue 21 to 100, it contains four α-helices named α1 to α4 (PDB ID 1R6R^32^). The first 20 residues are not shown since they are intrinsically disordered in solution^32^. (**b**) Molecular dynamics simulation structure of DENV C^29^, which highlights the three main structure/dynamics regions: disordered N-terminal (blue, residues 1–22)^31,32^; flexible fold (red, residues 23–44)^26^; and conserved fold (green, residues 45–100)^26,32–34^. (**c**) Superimposed DENV C ^1^H-^15^N HSQC spectra at pH 6.0 (gray), 6.5 (blue), 6.75 (red), 7.0 (yellow), 7.25 (green) and 7.5 (black). Our approach requires only ^1^H-^15^N HSQC peak intensities data. (**d**) Zoom on a spectral region where all types of response to the pH variation are observed: peaks from L44, I59 and K74 vary in intensity and chemical shift; K7, A8 and K73 just vary in intensity; R100 just varies in chemical shift; and, V26, F84 and R90 neither vary in intensity nor in chemical shift. UCSF Chimera v1.9 software^54^ was used for protein structure visualization. The data agrees with the current understanding of flaviviruses C proteins structure/dynamics and biological activity^25–34,55^.

Here, based on the amide hydrogen exchange process^10–12,20–24^, we report a simple and fast method to gain information on solvent accessibility of each amino acid residue N-H group of a protein. Benefiting from our previous work^25–30^, we usesd dengue virus (DENV) capsid (C) protein as a model (Fig. 1a, PDB ID 1R6R)^26,31,32^ since it possesses three distinct structural regions (Fig. 1b and Table S1): the disordered N-terminal, the flexible fold and the conserved fold. The nomenclature “conserved fold” refers to a structurally persistent fold that was found in the flaviviruses capsid protein structures of Dengue, West Nile and, recently, Zika viruses (with PDB IDs, respectively, 1R6R, 1SFK and 5YGH)^26,32–34^. This approach employs a small pH variation, preserving DENV C overall structure and dynamics, which easily allows probing the backbone N-H groups’ solvent accessibility (using only ^1^H-^15^N HSQC peak intensities). The three DENV C structure/dynamics regions can be clearly distinguished via this method, which supports not only the current understanding of DENV C structure/dynamics properties, but also the use of this technique to access and differentiate solvent-exposed N-H groups in IDP regions. Moreover, since the solvent accessibility of each N-H group is related to its intramolecular H-bond pattern, our approach also informs on the secondary structure content. Importantly, this methodology is readily applicable to study other proteins structure and dynamics. To demonstrate this, we also tested the effect of varying the pH, in a similar 1.5 pH units interval, on the NMR ^1^H-^15^N HSQC intensities using another model system, the B1 immunoglobulin binding domain of streptococcal protein G (GB1)^35–38^. GB1 is particularly interesting as it possesses α-helical and β-sheet regions, complementing the approach. As described ahead, the method is readily applicable to this protein as well, supporting its use.

## Results

### Suitability of the protein to the pH-based variation approach used

Our approach is based on small variations of pH within an interval that does not lead to major protein conformational changes. DENV C (Fig. 1a,b) was used as a model to study the relationship between the protein structure/dynamics and backbone N-H solvent accessibility (*i*.*e*., the ability of the N-H hydrogen to exchange with water hydrogens). The interval used is between pH 6.0 and 7.5, a physiological range that is suitable for most proteins, including DENV C, as described ahead. Taking advantage of the pH dependent amide N-H hydrogen exchange process^10–12,20–24^, ^1^H-^15^N HSQC spectra of DENV C were acquired at pH 6.0 and 7.5 (Fig. 1c,d, gray and black, respectively). Only specific peaks show decreased intensity and/or a chemical shift variation (Fig. 1c,d). The variations in intensity affect more peaks and are more pronounced than chemical shift variations, suggesting no major conformational change triggered by pH. To ascertain that, we acquired ^1^H-^15^N HSQC spectra of DENV C at several pH values (6.0, 6.5, 6.75, 7.0, 7.25 and 7.5), assessing the spectral evolution as a function of pH (Fig. 1c,d). The spectral region represented in Fig. 1d shows the four amino acid peaks that present the most pronounced variation of chemical shift, namely L44, K73, K74 and R100. Even for these residues, the changes are minimal, implying the conservation of DENV C overall architecture.

We then consulted the p*K*_a_ values of titratable residues (Fig. S1)^39^, since acid-base equilibrium could cause conformational changes that would difficult the interpretation of the results. DENV C theoretical isoelectric point is at pH 12.6 and, importantly, its sequence does not contain amino acid residues titratable within the pH range studied (Fig. S1, orange bar). We also measured the NMR transverse ^15^N amide relaxation rates (R_2_) in both pH conditions (Fig. S2), which demonstrated that there are no conformational transitions triggered by pH. This parameter is sensitive to alterations on the size/shape of a protein (since, in globular proteins, it generally increases with the protein hydrodynamic diameter), as well as to local fluctuations in the flexibility of particular amino acid residues^7,9,40–42^. It is clear from Fig. S2 that the R_2_ values obtained for DENV C are overall invariant in this pH range. Therefore, taking all of the above into account (namely Figs. 1c,d, S1 and S2), the overall DENV C structural arrangement is maintained. Thus, the pH-induced ^1^H-^15^N HSQC spectral differences are solely due to amide hydrogen exchange with water^10–12,20–24^, which reports on solvent accessibility.

### Probing solvent accessibility to the protein backbone

The spectral changes observed are consistent with an amide hydrogen exchange process (*i*.*e*., where N-H groups exchange their hydrogens with water hydrogens)^10–12,20–24^. Such process only occurs if N-H groups are exposed to the solvent and not in an intramolecular hydrogen bond. Therefore, these changes directly report on N-H groups’ solvent accessibility. At constant temperature, this exchange process occurs at a rate that increases 10 fold *per* pH unit^10–12,20–24^. Thus, here, by increasing the pH from 6.0 to 7.5, the hydrogen exchange rate constants increase 31.6 fold (*i*.*e*., 10^(7.5−6.0)^). This causes a decrease of the NMR peak intensity, since the fact that the amide proton starts to jump more frequently back and forth between the water and the amide sites leads to an enhanced decay of the transverse magnetization during acquisition. Spectral changes are therefore dependent on the extent of the increase of the N-H exchange rate constant. For the most solvent accessible N-H groups, peaks may even disappear from the spectrum at pH 7.5 (*e*.*g*., residues K7, A8 and L44 in Fig. 1d). These spectral changes can be highly informative if properly explored, reporting on structural and dynamic properties of proteins. As such, we studied them here, to develop a method that provides insights into protein structure and function, both at the individual amino acid and domain level.

**Figure 2:**
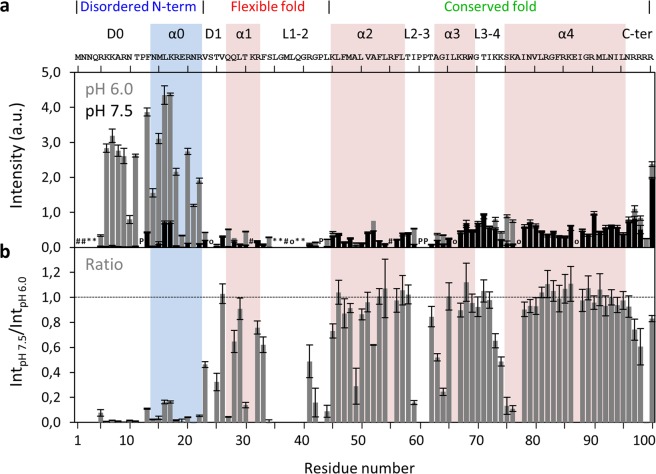
DENV C ^1^H-^15^N HSQC peak intensities at pH 6.0 and pH 7.5, and their ratio. (**a**) DENV C ^1^H-^15^N HSQC peak intensities at pH 6.0 (gray bars) and pH 7.5 (black bars), and (**b**) ratio of HSQC peak intensities at pH 7.5 and pH 6.0 (Int_pH 7.5_/Int_pH 6.0_). Error bars represent standard error (SE). The symbols in **a** encode the reason why the respective residues could not be analyzed by NMR: ‘#’ for residues that are not assigned, ‘o’ for overlaps, ‘*’ for absent resonances due to line broadening, and ‘P’ for prolines. The horizontal line on **b** marks the ratio equal to 1. The main structural features are indicated on the top of the figure: the three structure/dynamics regions^26,31,32^, the secondary structure domains^29,31,32^, and the protein primary sequence. Colored columns are a guide for the data corresponding to each secondary structure domain (pink columns represent the experimentally determined α-helices^31,32^, while the cyan column corresponds to the transient α-helix suggested by our previous work^29^).

To establish this new methodology, we first compared the maximum variation of pH values tested, by plotting the intensities at pH 6.0 and 7.5 as a function of protein sequence (Fig. 2a, gray and back, respectively). At pH 6.0, the N-H groups of N-terminal region residues display higher peak intensities, consistent with their disordered nature^26,31,32^. To simplify the analysis and compensate for differences in initial intensity (Int_pH 6.0_), results were normalized by the ratio between the intensities at pH 7.5 and 6.0 (Int_pH 7.5_/Int_pH 6.0_; Fig. 2b). The whole N-terminal region and specific residues located in the α1 and near loop regions decrease their intensity as the pH increases to 7.5. These findings are worth considering in the context of DENV C three main structural regions (Fig. 1b and Table S1). Briefly, at pH 7.5, the peak intensity of some residues is less than half of their initial values (Fig. 2b), namely: R5 to R22 (except P12) in the N-terminal region; V23 and T25 in the D1 domain; Q27 and T30 in α1; S34, R41, G42 and L44 in L1-2; A49 in α2; I59 and G64 near L2-3; K74, S75 and K76 near L3-4; and, R99 in the C-terminal domain. As such, the three main structure/dynamics regions of DENV C are clearly distinguishable, as further detailed ahead.

### Average solvent accessibility of protein regions

Given the above, we then analyzed the intensity changes in the context of the protein structure and dynamics of the main regions of the protein. For such purpose and although each N-H group of an individual amino acid behaves differently in response to pH^10–12,20–24^, we considered that structural factors are more determinant and we averaged the backbone N-H group response to pH across regions. Those that are protected, either by being buried within the structure or within an intramolecular hydrogen bond, will not be affected by pH. The amino acids that are not protected will be responsive to pH within the pH range tested here. The ratios determined in Fig. 2b, when averaged across a protein region or domain, provide a single parameter to distinguish between structural and dynamics sections.

**Figure 3:**
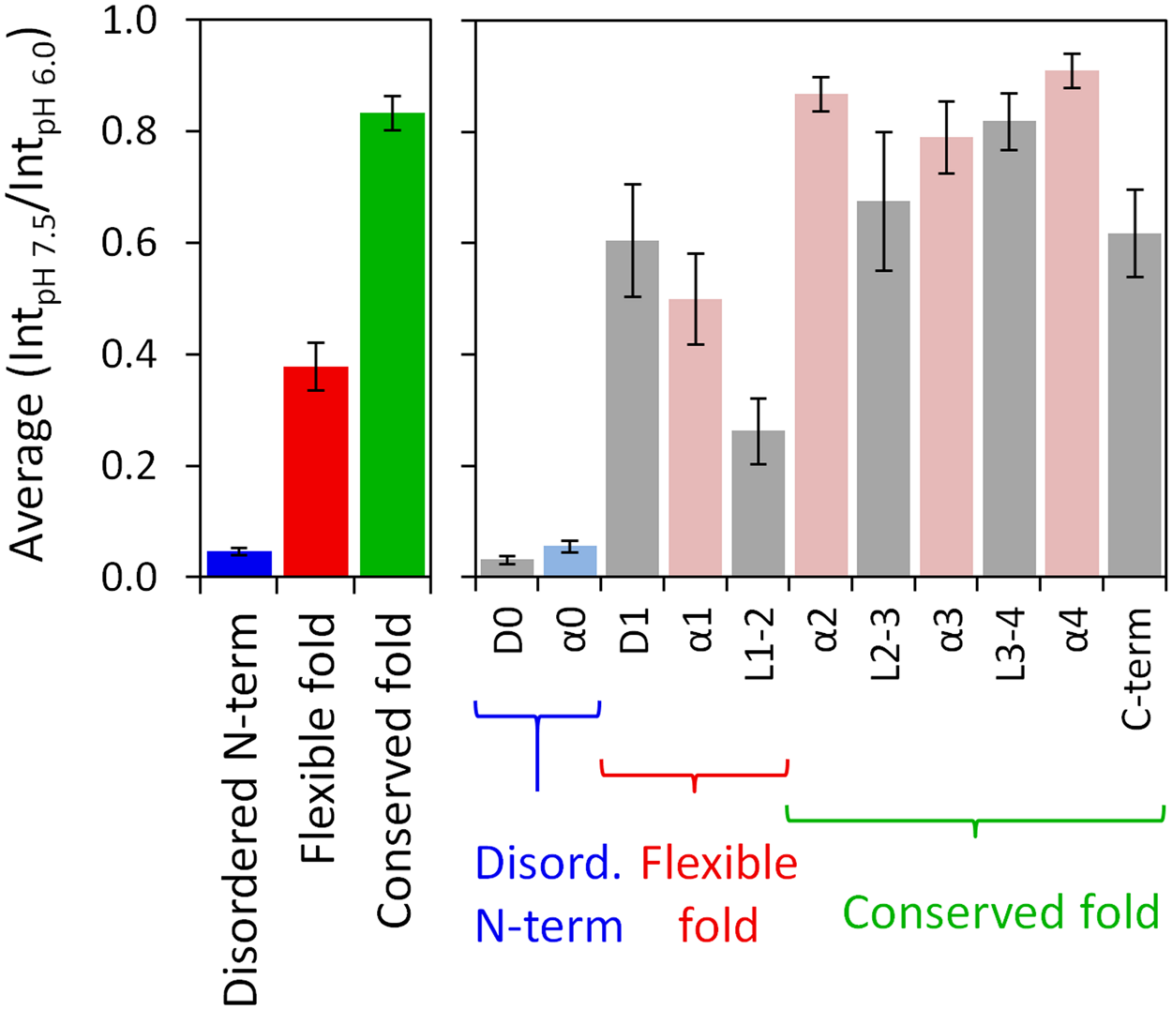
Average of the NMR peak intensities ratio for the three structure/dynamics regions and secondary structure domains of DENV C. The NMR peak intensities ratio between pH 7.5 and pH 6.0 for each residue (data from Fig. 2b) were averaged across the residues that comprise each of the three structure/dynamics regions (left panel) and secondary structure domains (right panel) of DENV C. Error bars are SE.

Fig. 3 depicts the average Int_pH 7.5_/Int_pH 6.0_ of the three main structure/dynamics regions (left panel) and of the secondary structure domains (right panel). The main structural regions are distinguished by their average backbone solvent accessibility (Fig. 3, left panel): the disordered N-terminal backbone is highly exposed, the flexible fold is partially accessible, and the conserved fold is mostly inaccessible to the solvent. Looking at the secondary structure domains (Fig. 3, right panel), the average values show that the backbone of the α0 domain, which is disordered and may transiently adopt an α-helical secondary structure^29^, is highly exposed to the solvent. Among the α-helices, the backbone of α1 presents values that are in between the obtained for α0 and those of the α2, α3 and α4 backbones, suggesting an intermediate exposure of α1 backbone to the solvent, implying a certain degree of flexibility. Therefore, DENV C α1 has more freedom to interact with the solvent, in line with our previous studies^26,29^. Moreover, the average backbone values for loop regions L2-3 and L3-4 are, in general, similar to those of nearby α-helices (Fig. 3, right panel). Thus, this approach probes the differences in backbone N-H groups solvent accessibility for the main structure/dynamics regions (Fig. 3, left panel), as well as for the secondary structure domains (Fig. 3, right panel).

### ^1^H-^15^N HSQC peak intensities in function of pH

**Figure 4:**
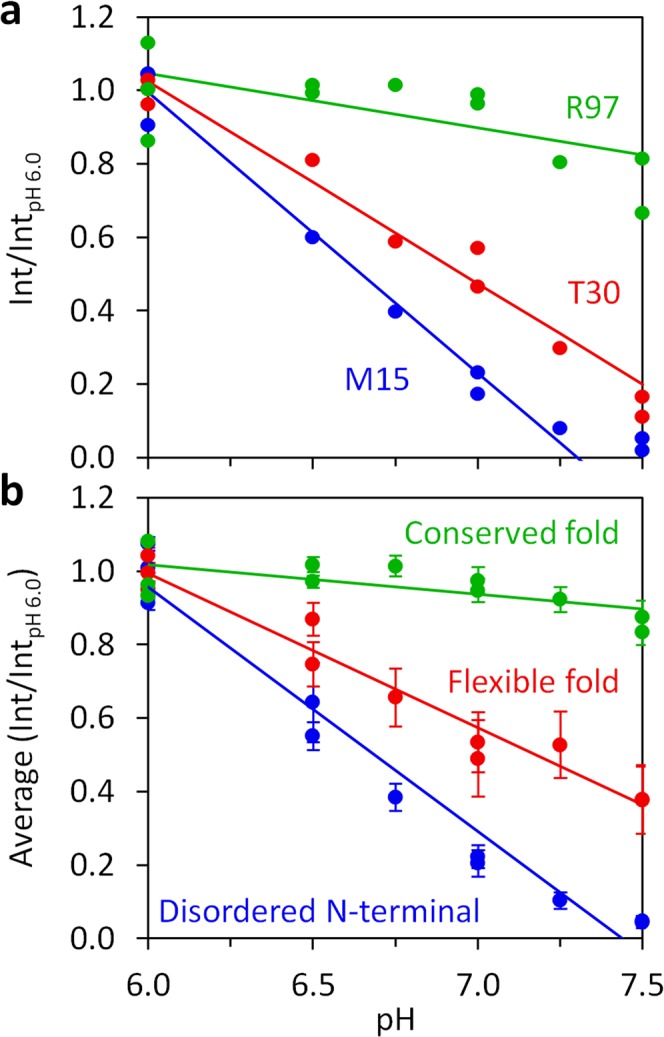
Normalized peak intensities of individual N-H groups and the average among DENV C structure/dynamics regions, as a function of pH. (**a**) Int/Int_pH 6.0_ variation with pH for the backbone N-H groups of M15, T30 and R97, representing residues in the disordered N-terminal, flexible fold and conserved fold regions, respectively. (**b**) Average intensities ratio for the three major structure/dynamics regions of DENV C. In all graphs, lines are fits of equation 1 to the data, from which slopes were extracted.

The changes in the ^1^H-^15^N HSQC spectrum for each pH tested (6.0, 6.5, 6.75, 7.0, 7.25 and 7.5) were assigned to the respective individual N-H groups of the protein (Fig. 1c,d), to give a complete picture of the peak evolution with pH. Fig. 4a shows the evolution of ^1^H-^15^N HSQC peak intensities as a function of pH for three residues (M15, T30 and R97) representative of the three main structural regions. Importantly, the solvent accessibility probed via the approach presented reports the interaction of each specific amide group with water. It can be used to distinguish the solvent accessibility of backbone and side-chain N-H groups within the same residue, as shown for W69 N-H groups (Fig. S3), where the backbone amide is not affected by pH, while the indole N-H group value varies significantly. Therefore, each N-H group reports its own microenvironment. The highly localized probing sensitivity illustrates the methodology great resolution level, a property that can be exploited to gain vital structural and dynamics information.

Besides this, we can analyze the normalized intensity of the backbone N-H groups, as a function of pH, for each amino acid, with striking differences between amino acids from different regions of the protein (Fig. 4a). An average of the normalized intensity of all the amino acids for each main region can then be obtained (Fig. 4b). The three key structural regions of DENV C are clearly distinguished (Fig. 4b): the conserved fold suffers no major changes (green), the N-terminal suffers the greatest change (blue), while the flexible fold shows an intermediate regime (red). The flexible fold also has larger error bars (Fig. 4b, red), indicative of higher heterogeneity among the constituting residues solvent accessibility. For each secondary structure domain, the average intensities as a function of pH are available in Fig. S4. Since the α0 domain^29^ is mostly disordered in solution^26,31,32^, its backbone average solvent accessibility is higher than for other α-helical domains, as expected. Importantly, α1 displays an intermediate accessibility and the other α-helical domains backbones are generally not exchanging the amide hydrogen with the solvent, in agreement with Fig. 3 data. Regarding loop domains, D0 and L1-2 have their backbone N-H groups mostly interacting with the solvent, while other loops are roughly unable to perform amide hydrogen exchange, in accordance with the analysis of Fig. 3. Therefore, we can obtain a single parameter that describes individual and regional exposure to the solvent, as described hereafter.

### Linearity of intensities *versus* pH

The backbone N-H peak intensities of individual amino acid residues follow a roughly linear decrease with pH (Fig. 4a), which is also observed for the average of the main regions (Fig. 4b) and domains (Fig. S4). As such, an approximation was used by fitting the following empirical linear equation to the data:

1 $$\frac{{\rm{Int}}}{{{\rm{Int}}}_{\mathrm{pH}6.0}}\approx {\rm{Slope}}\times ({\rm{pH}}-6.0)+1$$

where Int is the ^1^H-^15^N HSQC peak intensity at a given pH and Int_pH 6.0_ is the average intensity from 3 independent measurements at pH 6.0. The fitting of this equation retrieves the slope, which is a parameter that describes the average value of the derivative *d*(Int/Int_pH 6.0_)/*d*pH throughout the pH interval probed. A formal approach was also devised based on the literature^10–12,20–24^, which can be found on the Supplementary Note (of the Supplementary Information file), leading to the pH dependencies of both Int/Int_pH 6.0_ and *d*(Int/Int_pH 6.0_)/*d*pH. Importantly, this simpler slope-based (linear) approach retrieves a single fitting parameter that entirely describes the trend, being independent of external parameters estimation that sometimes are difficult to determine (*i*.*e*., k_rc_ or *t* values; for details, please consult the Supplementary Note). In practice, the more negative is the slope, the more susceptible to exchange is the corresponding N-H group. Slopes and Int_pH 7.5_/Int_pH 6.0_ values are comparable, as explained hereafter. Since slopes are originated from measurements at several pH values, they are a better parameter to represent each N-H group solvent accessibility, being of use to more advanced applications, and were employed henceforth.

### DENV C structure/dynamics and the slope information

**Figure 5:**
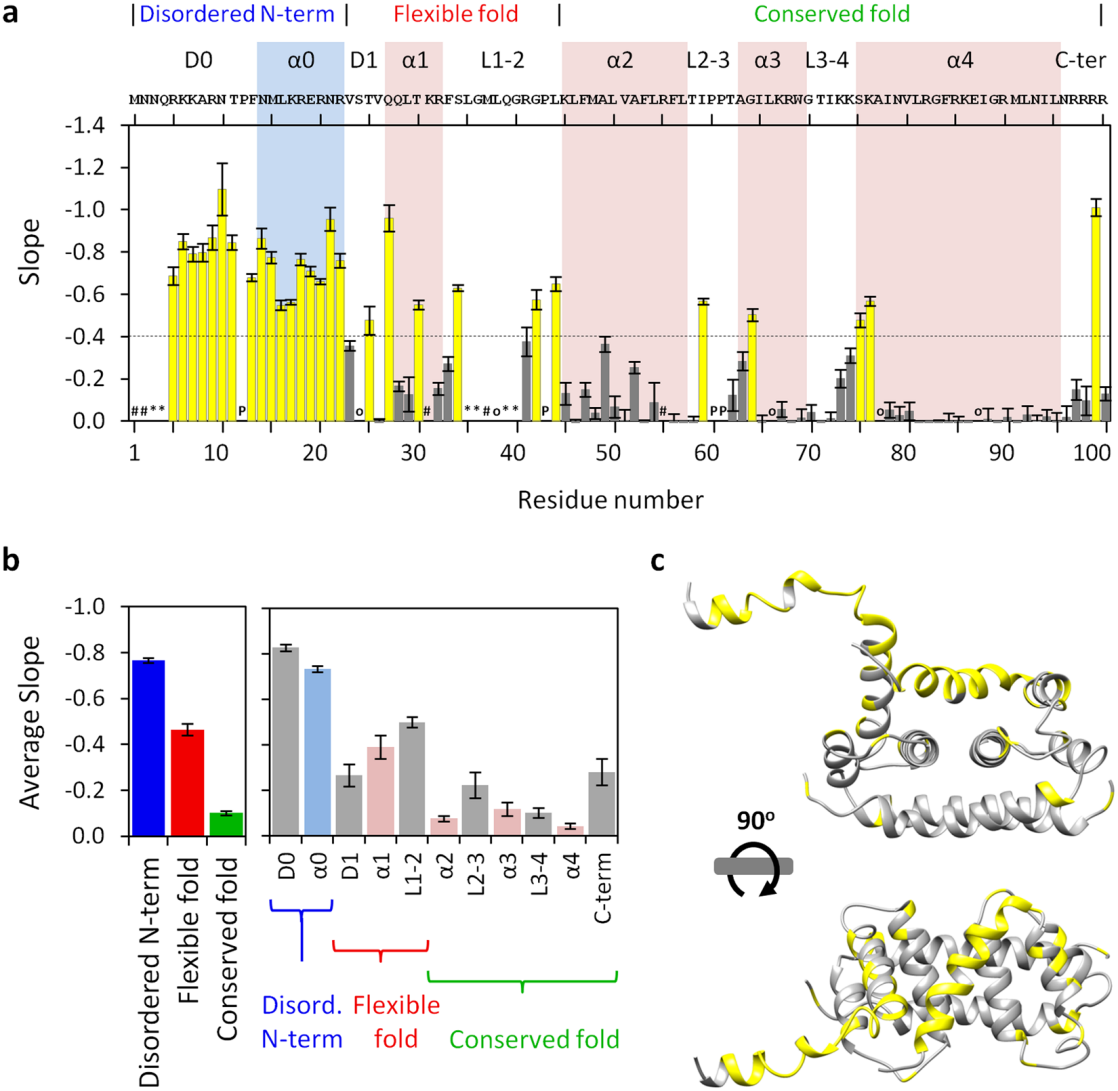
Slopes of Int/Int_pH 6.0_ *versus* pH in the context of the DENV C structure. (**a**) Slope of the intensities ratio *versus* pH along the DENV C sequence (an inverse scale is shown since the more efficient is the hydrogen exchange process, the more negative is the slope). The threshold of −0.4 (dashed line) was defined to identify the DENV C backbone N-H groups that are highly exposed to the solvent (yellow bars). For details on the protein structural information and symbols, on top and within the graph (respectively), please refer to the legend of Fig. 2. (**b**) Average slopes of the three structure/dynamics regions (b, left panel) and of the secondary structure domains (b, right panel) of DENV C. Error bars in **a** and **b** represent SE. (**c**) DENV C residues in which the backbone N-H is highly exposed to the water were highlighted within the protein structure (yellow regions). Clearly, from **a** and **c**, all the residues of the disordered N-terminal region, some specific residues on the flexible fold region and residues in the beginning of the α-helices are able to exchange their backbone amide hydrogen with the water.

Slope values were calculated via equation 1 for each analyzable DENV C backbone N-H group (Fig. 5a). The average slope values of the three major regions and of the secondary structure domains (Fig. S4) were then computed (Fig. 5b). The information obtained is similar to the one derived from the Int_pH 7.5_/Int_pH 6.0_ values (compare Figs. 5a and 2b, and also Figs. 5b and 3). In Fig. 5a, it is easy to distinguish the individual N-H groups that are fully exposed, intermediately exposed or buried away from the solvent. This is also clear in Fig. 5b for the three main regions and the several secondary structure domains. Noteworthy, within a given region, interconnecting loops seem to be more dynamic and exposed than adjacent α-helical domains, in consonance with the protein structure. Slope values of 0 (Fig. 5a) are from N-H groups of residues that cannot change their hydrogen with the solvent (corresponding to Int_pH 7.5_/Int_pH 6.0_ values of 1, in Fig. 2b). Slopes with absolute value higher than 0.7 (Fig. 5a) arise from N-H groups which are performing H-bonds with the water (corresponding to Int_pH 7.5_/Int_pH 6.0_ values of 0, in Fig. 2b). A slope threshold of −0.4 distinguishes the more solvent exposed N-H groups (Fig. 5a, yellow) from those less exposed (Fig. 5a, gray). A detailed analysis of Fig. 5a using this threshold reveals that the most solvent accessible N-H groups are from residues R5 to R22, T25, Q27, T30, S34, G42, L44, I59, G64, S75, K76 and R99. These residues are accordingly depicted in the protein structure (Fig. 5c, matching yellow and gray residues), providing direct information on both IDP and ordered regions of DENV C protein, which are immediately distinguishable. Moreover, many of these residues are located at the beginning of all the protein α-helices, which gives information on protein structure.

The fact that many of the first α-helical residues have N-H groups exposed to the solvent is a direct insight into the nature of α-helices. In an α-helix, the first residue is establishing H-bond via its C=O group with the N-H group of the fourth residue, leaving its own N-H group free of H-bonds involved in the α-helix stabilization. This means that the N-H groups of the first three residues of α-helices are free to establish H-bonds with other nucleophile groups (that serve as hydrogen bond acceptors) either from the protein, becoming unavailable to the solvent, or from the solvent. If they are exposed to the water, their amide hydrogen can exchange with those from the solvent. This is exactly what we observe in DENV C α-helices, by analyzing the backbone N-H groups performing intramolecular H-bonds within the DENV C structure (Fig. S5a). We then compared the slopes information with the normalized frequency of intramolecular H-bonds *per* N-H group (Fig. S5b), finding a clear correlation of the slopes with DENV C structure. Interestingly, residues that have low frequency of N-H intramolecular H-bonds (<0.5) and low slope values (between −0.4 and 0) are thus free to perform hydrogen exchange, but are unable to do so. This suggests that they are not facing the solvent because they are buried within the protein. In summary, an N-H group from a specific residue needs to be both free of intramolecular H-bonds and exposed to the solvent in order to exchange its hydrogen with the water. Overall, our findings suggest that the probing of the N-H groups’ solvent accessibility of a protein, via minor pH changes, may be used as an additional structure and dynamics restraint to help on the calculation of protein structures.

### Applying the method to GB1 protein

**Figure 6:**
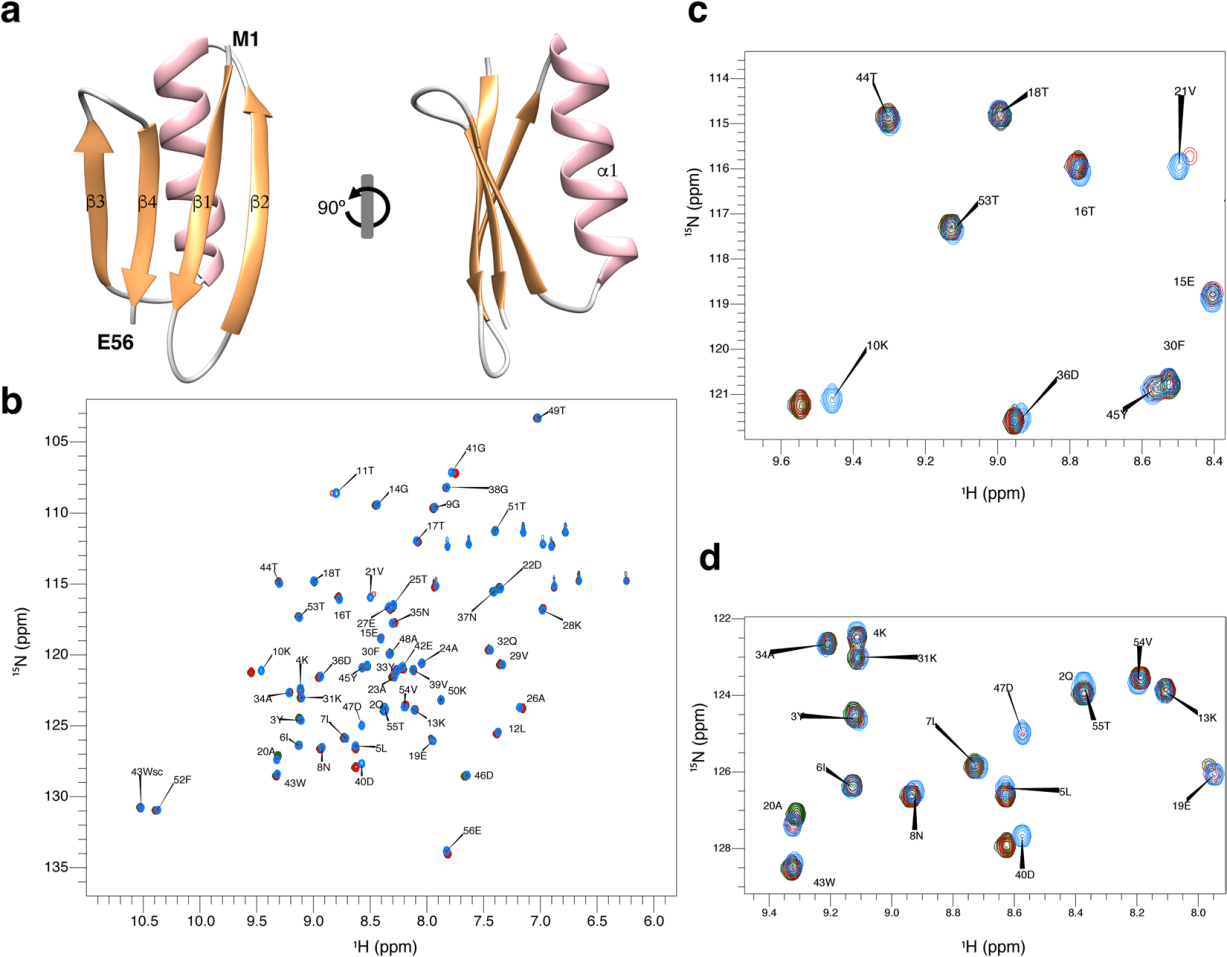
GB1 structure and ^1^H-^15^N HSQC spectra from pH 6.5 to 8.0. (**a**) GB1 experimental structure. GB1 is a domain of immunoglobulin G binding protein that is negatively charged, with 6 cationic and 10 anionic residues out of 56. It consists of four β-sheets, named β1 to β4 (colored in orange), plus one α-helix, named α1 (colored in pink), connected by short loops (PDB ID 5JXV^43^). (**b**) Superimposed GB1 ^1^H-^15^N HSQC spectra at pH 6.5 (blue), 7.3 (red), 7.6 (green) and 8.0 (black). Major chemical shifts corresponding to large conformational rearrangements are not observed. (**c** and **d**) Zoom on spectral regions where all types of responses to the pH variation are observed: peaks that vary in intensity and/or display a small chemical shift perturbation. Most peaks neither vary in intensity nor in chemical shift. UCSF Chimera v1.9 software^54^ was used for protein structure visualization^43^.

**Figure 7:**
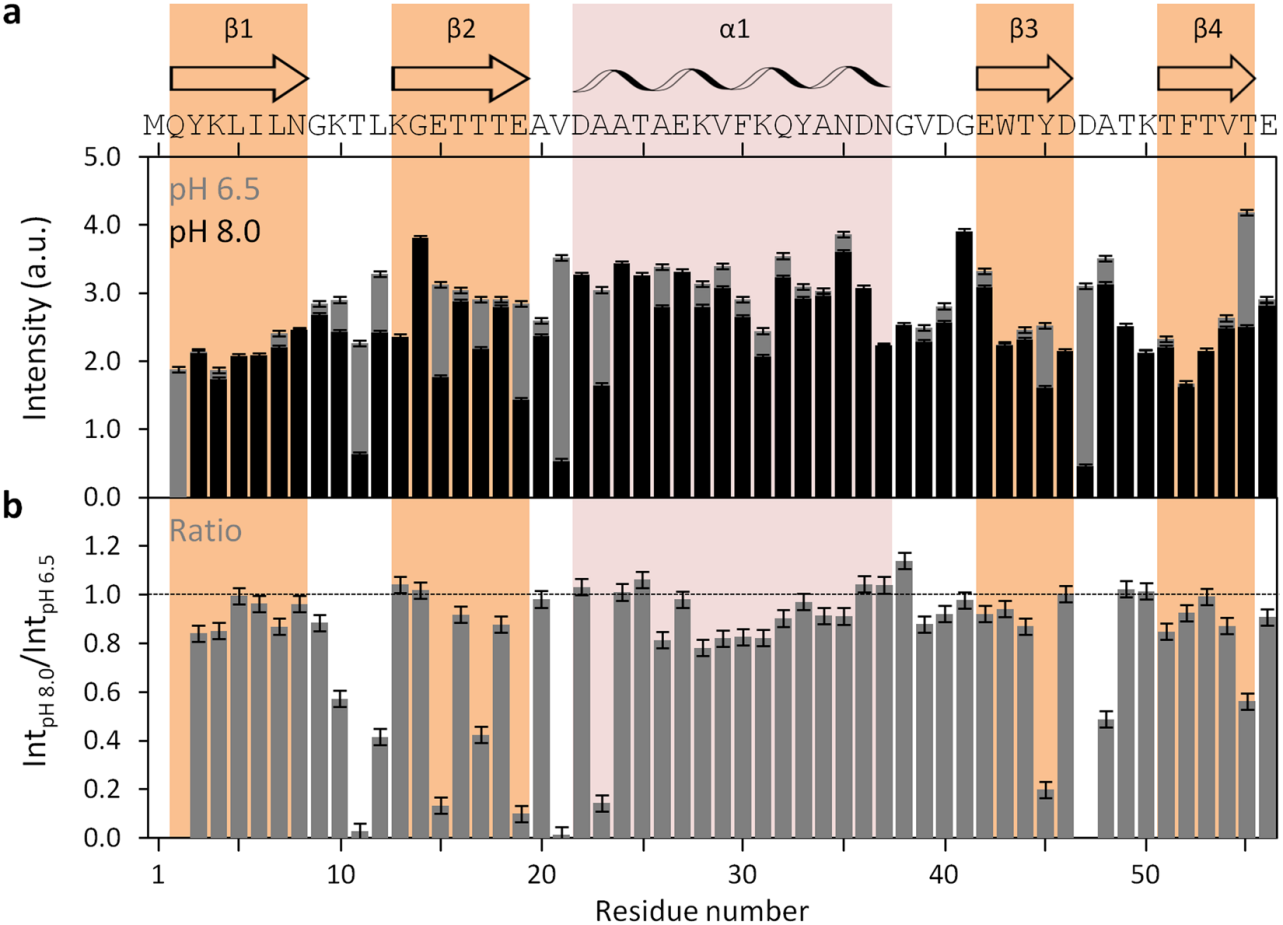
GB1 ^1^H-^15^N HSQC peak intensities at pH 6.5 and pH 8.0, and ratio between peaks. (**a**) GB1 ^1^H-^15^N HSQC peak intensities at pH 6.5 (gray bars) and pH 8.0 (black bars), and (**b**) ratio of HSQC peak intensities between pH 8.0 and pH 6.5 (Int_pH 8.0_/Int_pH 6.5_). Error bars represent standard error (SE). The M1 residue is not assigned, while Q2 was not analyzed due to absent resonance, as a result of line broadening. All other amino acid residue peaks were assigned and analyzed. The horizontal line on **b** marks the ratio equal to 1. The main structural features are indicated on the top of the figure: the secondary structure domains^36^, and the protein primary sequence. Colored columns are a guide for the data corresponding to each secondary structure domain (orange and pink columns correspond to experimentally determined β-sheets and α-helix, respectively.

Having established the method applicability with DENV C, we proceeded to test it with the B1 immunoglobulin binding domain of streptococcal protein G (GB1), which contains 56 amino acid residues and a structure of four stranded β-sheets with one long α-helix on top (Fig. 6a), as shown by X-Ray diffraction crystallography as well as by NMR (PDB ID: 2GB1 and 5JXV)^35,36,43^. GB1 has been extensively studied by different biophysical methods and is one of the smallest stable folded globular domains known. A pH interval of 1.5 was assayed as well, but now changing the pH from 6.5 to 8.0. No major conformational changes were seen (Fig. 6b), only minor local switches (Fig. 6c,d). As the overall protein structure remains highly stable within that pH range^37^, we went further and tested if the intensities of the ^1^H-^15^N HSQC peaks revealed any changes (Fig. 7). As for DENV C (Fig. 2), by directly comparing peak intensities at pH 6.5 and 8.0 on GB1 (Fig. 7a) or the ratio between these intensities (Fig. 7b), the major regions of the protein with exposed backbone amide nitrogen atoms can be readily identified, namely the loops, the outer strands of the four-stranded β-sheet (*i*.*e*., β2 and β3) and the beginning of the α-helix, which are free of backbone intramolecular H-bonds and accessible to the solvent.

**Figure 8:**
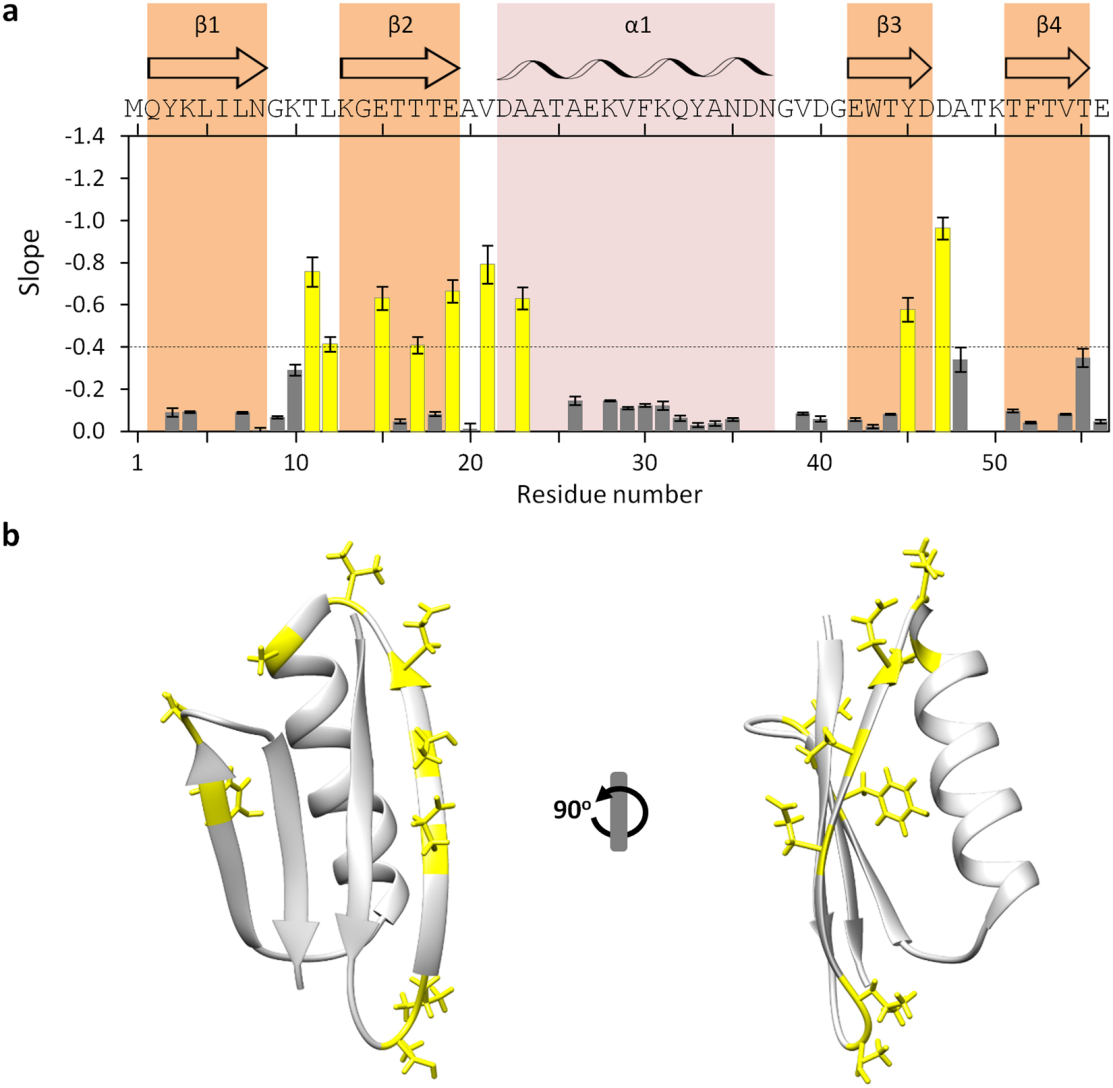
Slopes of Int/Int_pH 6.5_ *versus* pH in the context of the GB1 structure. (**a**) Slope of the intensities ratio *versus* pH along GB1 sequence (an inverse scale is shown since the more efficient is the hydrogen exchange process, the more negative is the slope). As in Fig. 5, the threshold of −0.4 (dashed line) was defined to identify GB1 backbone N-H groups that are highly exposed to the solvent (yellow bars). Error bars represent standard error (SE). Residues with positive slopes are not displayed (these are all close to zero). The protein structural information and symbols are colored as described in Fig. 7 legend. (**b**) GB1 residues in which the backbone N-H is exposed to the water were highlighted (yellow) within the protein structure, showing that only the amino acids exposed to the solvent (and not protected by intramolecular H-bonds) exchange their backbone amide hydrogen with the water.

Then, with the above in mind, we tested the use of the slope to map the protein regions most accessible to the solvent (Figs. 8 and S6), using the same cut-off as for DENV C. The information fits well with the known pattern of GB1 solvent-accessible surface area (SASA) along the sequence^44^, supporting the methodology employed. Moreover, our results are in accordance with H/D exchange studies, as the same regions identified as being more accessible to exchange with the solvent are the most sensitive to pH^38^. More protected residues that exchange through a global unfolding mechanism (*e*.*g*., residues K4, L5, A26, F30 and T44) or a local high energy unfolding mechanism (*e*.*g*., residues K28, Y33, N35 and T55) display minimal changes with pH, while the regions that correspond to fast exchanging non H-bonded N-H groups are clearly visible (*e*.*g*., residues T17, E19 and V21). This is also supported by the intramolecular H-bonds frequency analysis (Fig. S6), similarly to DENV C (Fig. S5). All this information further validates the methodology employed and suggests its applicability in other studies, as discussed ahead.

## Discussion

Taking advantage of the amide hydrogen exchange with water^10–12,20–24^, we established a new NMR approach to determine protein backbone solvent accessibility. Solvent accessibility correlates with the general structure/dynamics regions of a protein, with the explanation being straightforward: in more dynamic regions the N-H groups are more susceptible to hydrogen exchange with the solvent, as they are not involved in stable secondary structure elements. We may thus probe solvent accessibility by slightly varying the pH of a protein solution, as demonstrated by the study with DENV C, where a 1.5 pH units interval was employed (pH 6.0 to 7.5). The main protein regions (Fig. 1b) can be clearly distinguished via their individual amino acids (Figs. 2b and 5a) and their averaged backbone solvent exposure (Figs. 3, 4b and 5b). We can discriminate between the exposed disordered N-terminal, the partially accessible flexible fold and the mostly inaccessible conserved fold, in accordance with the current understanding of DENV C properties^26,29,31,32^. Noteworthy, the flexible fold intermediate behavior, previously predicted^26^, is confirmed here (Figs. 3, 4b and 5b, red). Furthermore, at single residue resolution, the solvent accessibility of each N-H group clearly correlates with its normalized frequency of intramolecular H-bonds in DENV C structure (Fig. S5). Such correlation is specific for each secondary structure element (such as α-helices and β-sheets), depending on the relative position in the protein structure. The same approach was then applied to a different system, GB1, a well structured protein domain, the B1 domain of immunoglobulin protein G. We employed another physiological interval of 1.5 pH units, from 6.5 to 8.0 (Fig. 6). The data shows that also with this protein there are no major conformational changes. Moreover, the key regions of the protein are also readily identified (Fig. 7), namely at the single amino acid level (Fig. 8).

Therefore, the pH range tested (6.0–8.0) is particularly suitable since most proteins isoelectric point is not close to the physiological pH (since they would not be functional as they could precipitate) and, apart from histidines, titratable amino acids are usually not affected at this pH range, as inferred from Fig. S1. Even if minor conformational changes occur, these would be readily visible in the NMR spectra through chemical shift perturbation (CSP) studies, allowing it to be taken into account in the analysis. So, the methodology can be easily applied to other proteins, in pH ranges where their general structure and dynamics properties are maintained. After NMR assignment at low pH (*e*.*g*., pH 6.0), one can perform a pH increase on the same sample (at least one pH unit is recommended) and acquire one more ^1^H-^15^N HSQC spectrum to determine immediately which N-H groups and protein regions interact with the solvent. A single protein preparation (at concentrations around 5-10 mg/mL) can be used and two measurements at different pH values readily provide key information. Even if there is a mild conformational change triggered by pH and/or titration of some specific amino acid residue(s), a pH variation can be performed by acquiring ^1^H-^15^N HSQC spectra at several slightly spaced pH values, to follow the NMR peaks evolution (CSP analysis). In particular, the pH range used here (between 6.0 and 8.0) has the advantage of being physiologically relevant and compatible with the timescale of usual ^1^H-^15^N HSQC measurements.

Moreover, the approach is particularly useful to study IDP regions or when NMR data is sparse due to time or other constraints. As mentioned, the intensity of a signal depends on the line width, which is mainly influenced by the protein correlation time and the chemical exchange regime: sharp for IDPs (due to correlation time below 1 ns, but potentially broadened due to accessibility for water exchange) while amides in the folded part of a protein will experience less line broadening due to slower water exchange. Overall, if one changes the pH by 1.5 units, the base-catalyzed hydrogen exchange increases by 10^1.5^, which has large effects on the intensity (the reciprocal line width) of the IDP signals and a smaller effect on the folded parts, as readily observed here. Other methodologies have been described to improve protein structure determination, namely residual dipolar couplings (RDCs)^14,17^, diffusion tensor parameters^15^, relaxation parameters^16^ and paramagnetic relaxation enhancement (PRE) probes^17^, among others. Nevertheless, since the method described here is much simpler and easier to interpret, we believe that it will be widely adopted for protein structural and dynamics studies.

Beyond the methodological development, the analysis employed in this study also gives important information on the DENV C structure/dynamics properties and their functional implications. From this analysis it is clear that the three major regions are directly observable and that the α-helices identified have different dynamic properties. Briefly, α0 region is more malleable than the other secondary structure domains and α1 is partially flexible when compared to α2, α3 and α4. The N-terminal IDP region as well as the first amino acids of the α-helices and the loop domains are accessible to the solvent, in agreement with the available DENV C structure (PDB ID 1R6R^32^). Overall, the results are consistent with the current understanding of DENV C structure and function^25,26,28–32^, supporting the use of the proposed methodology to investigate protein architecture.

Importantly, the findings concerning the flexible fold region are particularly new and shed a new light on the protein biological activity. The term “flexible fold”, to refer to that section of the protein, was first used by us upon comparing DENV C NMR structure with the capsid protein X-Ray crystallographic structure of the closely related West Nile virus^26,32,33^. At that time, it only meant that this section, which contains an α-helix in both C proteins, was not folded in a similar manner in the context of the homodimer. Very recent studies showed the same when comparing with the Zika virus C protein structure^34^. Nevertheless, only now did it become evident that in DENV C this section is much more free to interact with the solvent. This, alongside with the N-terminal IDP region, may modulate an auto-inhibitory mechanism that regulates the solvent access to the hydrophobic interface region previously proposed by us^29^. This hydrophobic pocket is essential for DENV C binding to lipid droplets and, subsequently, for viral replication. Therefore, by targeting this region, such understanding may help to improve and design new drugs against dengue virus and related flaviviruses.

In addition, our studies with GB1 (Figs. 6, 7 and 8) fully demonstrate that those regions and amino acid residues most exposed to the solvent are easily distinguished. The data also correlate well with the intramolecular H-bond pattern (Fig. S7). In short, the method is applicable to different proteins and can complement, in a faster way, present NMR experimental routines.

To conclude, while gaining important information on a key viral protein, we also report a fast NMR-based method to determine the protein backbone solvent accessibility at single amino acid N-H group resolution, which is applicable to other proteins besides DENV C, as demonstrated here with GB1. The approach is of special interest to study IDP regions, where classical techniques are difficult to employ. Moreover, the method is applied in a physiologically relevant pH range, providing valuable insights into protein structure and function. This work provides the basis to further studies, where the application of small physiological pH changes to interrogate protein structure, dynamics and solvent accessibility is conducted. These can be performed in conditions suited for the analysis of IDP and structured regions, complementing the array of methodologies available for protein studies, particularly of IDP regions that are difficult to assay via current techniques.

## Methods

### Materials

Isotopically enriched chemical compounds, namely [^15^N]-NH_4_Cl, [^13^C]-glucose and ^2^H_2_O, were purchased from Cambridge Isotope Laboratories (Tewksbury, MA, USA). Chromatography columns HiTrap Heparin, HiTrap Q HP and Superdex 75 10/300 GL, the Peristaltic Pump P-1 and AKTA Start were from GE Healthcare (Little Chalfont, UK). Reagents for SDS-PAGE were from BioRad (Hercules, CA, USA). Dialysis membrane tube Spectra/Por of 3.5 kDa nominal cut-off was from Spectrum Laboratories (Los Angeles, CA, USA) and Amicon Ultra-4 or -15 centrifugal filters of 10 or 3 kDa nominal cut-off were from Millipore (Billerica, MA, USA). Unless otherwise described, all other chemicals were purchased from Sigma-Aldrich (St. Louis, MO, USA).

### DENV C – heterologous protein expression and purification

DENV C protein purification protocol was optimized from previous studies^25,26,28,31,32,45^. The protein was expressed in *E*. *coli* BL21-CodonPlus transformed with a pET-21a plasmid with a gene encoding the capsid protein of DENV serotype 2, strain New Guinea C (NCBI ID AAC59275, corresponding to amino acids 1–100 of the polyprotein)^25,26,28,46^. Further details are available as Supplementary Information, in the Supplementary Methods section.

### GB1 – heterologous protein expression and purification

The pET11a plasmid containing the gene encoding T2Q B1 immunoglobulin G binding domain of streptococcal protein G (GB1) was kindly provided by Professor Gary Pielak, from University of North Carolina at Chapel Hill. The T2Q mutation prevents N-terminal deamidation. This form is mentioned here as wild type (WT) or only “GB1”. The isolation and purification of ^15^N,^13^C enriched GB1 was optimized from previous studies^35–37,44^. Further details are available as Supplementary Information, in the Supplementary Methods section.

### DENV C – pH variation, sample preparation and NMR experiments

Prior to the measurements, DENV C stock solution was diluted to 2/5 with a 55 mM KH_2_PO_4_, 13.75 mM NaN_3_, pH 6.0 solution and the protein concentration was adjusted to 550 μM (monomer), either by diluting or concentrating (with Amicon Ultra-4 Centrifugal Filters of 10 kDa cut-off) with a solution of 55 mM KH_2_PO_4_, 220 mM KCl and 5.5 mM NaN_3_, pH 6.0. In order to change the pH in a controlled way in small volumes, we performed a dialysis of 500 μL of protein solution, containing 550 μM of DENV C, 55 mM KH_2_PO_4_, 220 mM KCl and 5.5 mM NaN_3_, pH 6.0, in an uncapped eppendorf tube sealed with a Spectra/Por dialysis membrane of 3.5 kDa cut-off, which was then tightly sealed with Parafilm. Each eppendorf tube was put upside down in a different flask containing 100 mL (to be 200 × 500 μL) of the same buffer solution in which the protein is dissolved in, but with different pH value. These dialyses were performed at room temperature at least for 1 h before the experiment. After dialysis and a quick spin-down centrifugation of the eppendorf tubes, ^2^H_2_O was added to a 10% (v/v) final concentration and the solution was transferred to a standard NMR tube. In every measurement, we checked the final pH within the NMR tube (after ^2^H_2_O addition), which matched the expected pH. The final solutions contained 500 μM of DENV C monomer, 50 mM KH_2_PO_4_, 200 mM KCl, 5 mM NaN_3_ and 10% (v/v) ^2^H_2_O, at different pH values: 6.0, 6.5, 6.75, 7.0, 7.25 or 7.5. NMR peak intensities are normalized to the average intensity at pH 6.0 (Int/Int_pH 6.0_).

We performed ^15^N transverse relaxation (R_2_) NMR experiments were performed at 298.15 K in a Bruker Avance III 800 MHz equipped with a triple resonance (^1^H, ^13^C, ^15^N) probe. Spectra were processed using NMRPipe^47^ and analyzed with NMRViewJ^48^. These spectra were acquired as pseuso-3D, with 2D ^1^H-detected, ^15^N-edited HSQC experiments, implementing standard pulse sequences^40–42,49^. R_2_ spectra were recorded with spectral widths of 1024 × 256 complex points in the ^1^H and ^15^N dimensions, respectively. The field strength of the CPMG refocusing train was 500 Hz and a 1.2 ms delay was used between the refocusing pulses^50,51^. The effects of cross relaxation between ^1^H-^15^N dipolar and ^15^N chemical shift anisotropy were removed by applying ^1^H 180° pulses during relaxation delays^52^. Further details are available in the Supplementary Information, Supplementary Methods section.

### GB1 – pH variation, sample preparation and NMR experiments

^13^C-^15^N GB1 was dissolved in 100 mM KCl buffer in 90% H_2_O/10% D_2_O containing 100 μM DSS (used as internal reference) to a protein concentration of 1 mM. The pH was measured using a Docu-pH meter (Sartorius) calibrated with standard solutions. The initial pH was 6.50. For each pH step, the pH was adjusted with microliter additions of 0.15 M NaOH solution. The concentration of added salt amounted to less than 1 mM. Spectra were collected in steps of ~0.4 pH units from pH 6.5 to 8.0. Data were processed using Bruker TopSpin^TM^ 4.0 and analyzed with CCPNMR^53^ for cross-peak assignment and height extraction. Further details are available in the Supplementary Information, Supplementary Methods section.

### Further experimental details

Further details are available as Supplementary Information, in the Supplementary Note and the Supplementary Methods sections.

## Supplementary information

Supplementary Information

## Data Availability

All data generated or analyzed during this study are included in this article and Supplementary Information file.

## References

[1] Cavanagh, J., Fairbrother, W. J., Palmer, A. G. I., Rance, M. & Skelton, N. J. *Protein NMR Spectroscopy – Principles and Practice*. Second Edition (2007).

[2] Raman S (2010). NMR structure determination for larger proteins using backbone-only data. Science.

[3] Korzhnev DM, Religa TL, Banachewicz W, Fersht AR, Kay LE (2010). A transient and low-populated protein-folding intermediate at atomic resolution. Science.

[4] Lange OF (2012). Determination of solution structures of proteins up to 40 kDa using CS-Rosetta with sparse NMR data from deuterated samples. Proc. Natl. Ac. Sci. USA.

[5] van der Schot G (2013). Improving 3D structure prediction from chemical shift data. J. Biomol. NMR.

[6] van der Schot G, Bonvin AM (2015). Performance of the WeNMR CS-Rosetta3 web server in CASD-NMR. J. Biomol. NMR.

[7] Mittermaier AK, Kay LE (2009). Observing biological dynamics at atomic resolution using NMR. Trends Biochem. Sci..

[8] Kay LE (2005). NMR studies of protein structure and dynamics. J. Magn. Reson..

[9] Kleckner IR, Foster MP (2011). An introduction to NMR-based approaches for measuring protein dynamics. Biochim. Biophys. Acta.

[10] Englander SW, Mayne L (1992). Protein folding studied using hydrogen-exchange labeling and two-dimensional NMR. Annu. Rev. Biophys. Biomol. Structure.

[11] Krishna MM, Hoang L, Lin Y, Englander SW (2004). Hydrogen exchange methods to study protein folding. Methods.

[12] Englander SW, Mayne L, Bai Y, Sosnick TR (1997). Hydrogen exchange: the modern legacy of Linderstrom-Lang. Protein Sci..

[13] Vendruscolo M, Paci E, Dobson CM, Karplus M (2003). Rare fluctuations of native proteins sampled by equilibrium hydrogen exchange. J. Am. Chem. Soc..

[14] Zeng J (2009). High-resolution protein structure determination starting with a global fold calculated from exact solutions to the RDC equations. J. Biomol. NMR.

[15] Ryabov Y, Suh JY, Grishaev A, Clore GM, Schwieters CD (2009). Using the experimentally determined components of the overall rotational diffusion tensor to restrain molecular shape and size in NMR structure determination of globular proteins and protein-protein complexes. J. Am. Chem. Soc..

[16] Ryabov Y, Schwieters CD, Clore GM (2011). Impact of ^15^N R_2_/R_1_ relaxation restraints on molecular size, shape, and bond vector orientation for NMR protein structure determination with sparse distance restraints. J. Am. Chem. Soc..

[17] Jensen MR, Zweckstetter M, Huang JR, Blackledge M (2014). Exploring free-energy landscapes of intrinsically disordered proteins at atomic resolution using NMR spectroscopy. Chem. Rev..

[18] Rossi P (2015). A hybrid NMR/SAXS-based approach for discriminating oligomeric protein interfaces using Rosetta. Proteins.

[19] Varadi M (2014). pE-DB: a database of structural ensembles of intrinsically disordered and of unfolded proteins. Nucleic Acids Res..

[20] Bai Y, Milne JS, Mayne L, Englander SW (1993). Primary structure effects on peptide group hydrogen exchange. Proteins.

[21] Connelly GP, Bai Y, Jeng MF, Englander SW (1993). Isotope effects in peptide group hydrogen exchange. Proteins.

[22] Bai Y, Sosnick TR, Mayne L, Englander SW (1995). Protein folding intermediates: native-state hydrogen exchange. Science.

[23] Koide S, Jahnke W, Wright PE (1995). Measurement of intrinsic exchange rates of amide protons in a 15N-labeled peptide. J. Biomol. NMR.

[24] Hwang TL, van Zijl PC, Mori S (1998). Accurate quantitation of water-amide proton exchange rates using the phase-modulated CLEAN chemical EXchange (CLEANEX-PM) approach with a Fast-HSQC (FHSQC) detection scheme. J. Biomol. NMR.

[25] Carvalho FA (2012). Dengue virus capsid protein binding to hepatic lipid droplets (LD) is potassium ion dependent and is mediated by LD surface proteins. J. Virol..

[26] Martins IC (2012). The disordered N-terminal region of dengue virus capsid protein contains a drug targetable lipid droplet-binding motif. Biochem. J..

[27] Martins, I. C., Almeida, F. C. L., Santos, N. C. & Da Poian, A. T. DENV-Derived Peptides and Methods for the Inhibition of the Flavivirus Replication, Universidade Federal do Rio de Janeiro (UFRJ), Universidade de Lisboa (UL), Instituto de Medicina Molecular (IMM). International Patent Publication Nr WO/2012/159187 (2012)

[28] Faustino AF (2014). Dengue virus capsid protein interacts specifically with very low-density lipoproteins. Nanomedicine: NBM.

[29] Faustino AF (2015). Understanding dengue virus capsid protein disordered N-terminus and pep14-23-based inhibition. ACS Chem. Biol..

[30] Faustino AF (2015). Understanding dengue virus capsid protein interaction with key biological targets. Sci. Rep..

[31] Jones CT (2003). Flavivirus capsid is a dimeric alpha-helical protein. J. Virol..

[32] Ma L, Jones CT, Groesch TD, Kuhn RJ, Post CB (2004). Solution structure of dengue virus capsid protein reveals another fold. Proc. Natl. Acad. Sci. USA.

[33] Dokland T (2004). West Nile virus core protein; tetramer structure and ribbon formation. Structure.

[34] Shang Z, Song H, Shi Y, Qi J, Gao GF (2018). Crystal structure of the capsid protein from Zika virus. J. Mol. Biol..

[35] Gallagher T, Alexander P, Bryan P, Gilliland GL (1994). Two crystal structures of the B1 immunoglobulin-binding domain of streptococcal protein G and comparison with NMR. Biochemistry.

[36] Gronenborn AM (1991). A novel, highly stable fold of the immunoglobulin binding domain of streptococcal protein G. Science.

[37] Lindman S (2006). Salting the charged surface: pH and salt dependence of protein G B1 stability. Biophys. J..

[38] Orban J, Alexander P, Bryan P, Khare D (1995). Assessment of stability differences in the protein G B1 and B2 domains from hydrogen-deuterium exchange: comparison with calorimetric data. Biochemistry.

[39] Toseland CP, McSparron H, Davies MN, Flower DR (2006). PPDv1.0–an integrated, web-accessible database of experimentally determined protein pKa values. Nucleic Acids Res..

[40] de Medeiros LN (2010). Backbone dynamics of the antifungal Psd1 pea defensin and its correlation with membrane interaction by NMR spectroscopy. Biochim. Biophys. Acta.

[41] de Paula VS (2013). Structural basis for the interaction of human beta-defensin 6 and its putative chemokine receptor CCR2 and breast cancer microvesicles. J. Mol. Biol..

[42] de Paula VS, Razzera G, Barreto-Bergter E, Almeida FC, Valente AP (2011). Portrayal of complex dynamic properties of sugarcane defensin 5 by NMR: multiple motions associated with membrane interaction. Structure.

[43] Andreas LB (2016). Structure of fully protonated proteins by proton-detected magic-angle spinning NMR. Proc. Natl. Acad. Sci. USA.

[44] Monteith WB, Pielak GJ (2014). Residue level quantification of protein stability in living cells. Proc. Natl. Acad. Sci. USA.

[45] Samsa MM (2009). Dengue virus capsid protein usurps lipid droplets for viral particle formation. PLoS Pathog..

[46] Irie K, Mohan PM, Sasaguri Y, Putnak R, Padmanabhan R (1989). Sequence analysis of cloned dengue virus type 2 genome (New Guinea-C strain). Gene.

[47] Delaglio F (1995). NMRpipe - a Multidimensional Spectral Processing System Based on Unix Pipes. J. Biomol. NMR.

[48] Johnson BA, Blevins RA (1994). NMR View: A computer program for the visualization and analysis of NMR data. J. Biomol. NMR.

[49] Farrow NA (1994). Backbone dynamics of a free and phosphopeptide-complexed Src homology 2 domain studied by 15N NMR relaxation. Biochemistry.

[50] Carr HY, Purcell EM (1954). Effects of diffusion on free precession in nuclear magnetic resonance experiments. Phys. Rev..

[51] Meiboom S, Gill D (1958). Modified spin-echo method for measuring nuclear relaxation times. Rev. Sci. Instrum..

[52] Palmer AG, Williams J, McDermott A (1996). Nuclear magnetic resonence studies of biopolymer dynamics. J. Phys. Chem..

[53] Vranken WF (2005). The CCPN data model for NMR spectroscopy: development of a software pipeline. Proteins.

[54] Pettersen EF (2004). UCSF Chimera–a visualization system for exploratory research and analysis. J. Comput. Chem..

[55] Martins AS (2018). Methods for Lipid Droplet Biophysical Characterization in *Flaviviridae* Infections. Front. Microbiol..

