# Supplementary material for: Fast NMR method to probe solvent accessibility and disordered regions in proteins
Source: Sci Rep. 2019 Feb 7;9:1647. doi: 10.1038/s41598-018-37599-z (PMC6367444; doi:10.1038/s41598-018-37599-z)
Supplement: Supplementary file 1 — Supplementary Information [file 41598_2018_37599_MOESM1_ESM.pdf]

SUPPLEMENTARY INFORMATION

TITLE

Fast NMR method to probe solvent accessibility and disordered regions in proteins

AUTHORS

André F. Faustino[#1], Glauce M. Barbosa[2], Micael Silva[4], Miguel A. R. B. Castanho[1], Andrea T. Da Poian[2], Eurico J. Cabrita[4], Nuno C. Santos[*1], Fabio C. L. Almeida[*2, 3], Ivo C. Martins[*1]

AFFILIATIONS

[1]Instituto de Medicina Molecular, Faculdade de Medicina, Universidade de Lisboa, Av. Prof. Egas Moniz, 1649-028 Lisbon, Portugal;

[2]Instituto de Bioquímica Médica Leopoldo de Meis, Universidade Federal do Rio de Janeiro, Rio de Janeiro, 21941-902 RJ, Brazil;

[3]Centro Nacional de Ressonância Magnética Nuclear, Universidade Federal do Rio de Janeiro and National Institute of Structural Biology and Bioimage (CENABIO), Rio de Janeiro, 21941-902 RJ, Brazil;

[4]REQUIMTE, UCIBIO, Departamento de Química, Faculdade de Ciências e Tecnologia, Universidade Nova de Lisboa, Quinta da Torre, 2829-516 Monte de Caparica, Portugal.

[*]To whom correspondence should be addressed.

E-mails: ivomartins@medicina.ulisboa.pt; falmeida@bioqmed.ufrj.br; nsantos@fm.ul.pt.

[#]Present address: iBET, Instituto de Biologia Experimental e Tecnológica, Apartado 12, 2780-901 Oeiras, Portugal.

SUPPLEMENTARY FIGURES AND TABLES

Table S1. DENV C main structure/dynamics regions and secondary structure domains.

Structure/dynamics	Secondary structure	First residue	Last residue
**disordered N-terminal** (residues 1-22)	D0	M1	F13
	α0	N14	R22
**flexible fold** (residues 23-44)	D1	V23	V26
	α1	Q27	R32
	L1-2	F33	L44
**conserved fold** (residues 45-100)	α2	K45	L57
	L2-3	T58	T62
	α3	A63	W69
	L3-4	G70	K74
	α4	S75	L95
	C-terminal	N96	R100

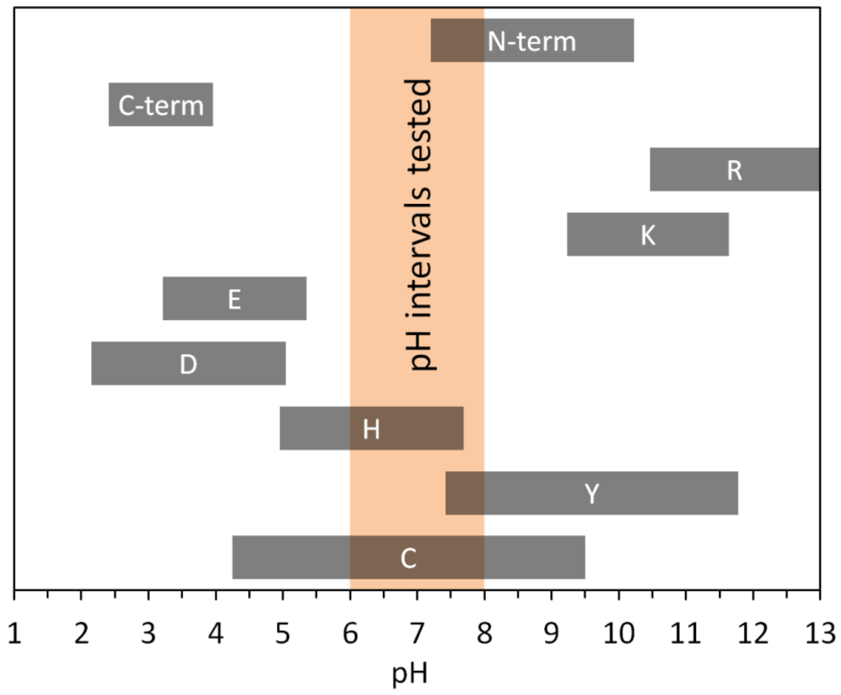

Figure S1. Usual pK_a values of the titratable amino acid side chains in proteins. Plot of the pK_a values experimentally measured for 163 proteins, corresponding to 1401 amino acid residue entries[1]. Rectangles are centered at the average value and half of their length corresponds to the standard deviation, SD. The SD of R is not available in the literature[1]and, thus, we plotted it as the average of the SD values for the other amino acids. The titratable groups of DENV C are: R (n=16), K (n=10), E (n=2), the N-terminus and the C-terminus. For GB1, the titratable groups are: K (n=6), E (n=5), D (n=5), the N-terminus and the C-terminus. The orange column represents the range of pH values tested in this study, suggesting that neither DENV C nor GB1 residues change the protonation state between pH 6.0 and pH 8.0. Although the effect of pH on DENV C and GB1 structure/dynamics properties is negligible (see Figs. 1 and 6), proteins with histidines or free cysteines at their surface (which is not very common) are more likely to be affected by pH. However, since the proposed analysis is based on NMR spectra, it can also be informative in those cases if the approach is complemented with chemical shift perturbation (CSP) analysis.

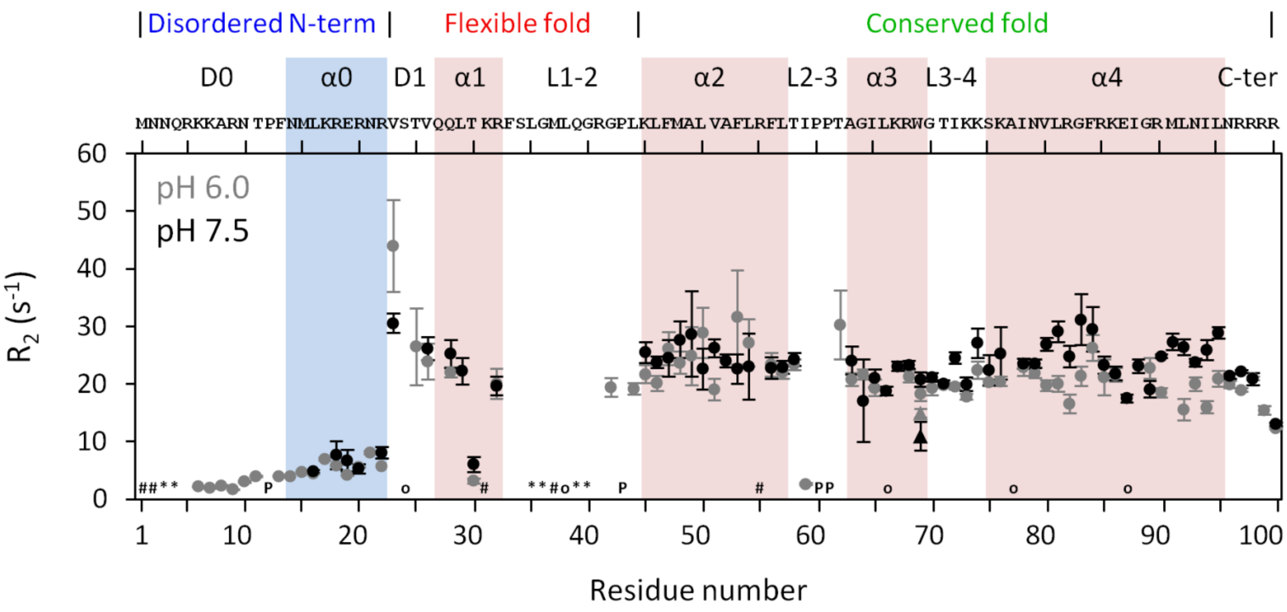

Figure S2. NMR ^{15}N transverse relaxation (R_2) of DENV C at pH 6.0 and 7.5. NMR ^{15}N R_2 values at pH 6.0 (gray circles) and at pH 7.5 (black circles) are generally similar. Triangles at position W69 represent the R_2 values of the W69 indole ^{15}N. For details on the protein structural information and symbols, on top and within the graph (respectively), please refer to the legend of Fig. 2. Error bars represent SE. Some exceptions to the general invariance of R_2 values occur within α4, where residues V80 to G83 and R90 to L95 increase their R_2 values at pH 7.5, relative to pH 6.0. Importantly, none of these α4 residues present variations of their NMR peak intensities within the pH range tested (compare with Fig. 2b of the manuscript). This indicates that the protein maintains its homodimer structure and, at a more local level, that the large majority of residues maintain their structure/dynamics properties in both pH conditions. This indicates that no conformational changes triggered by this pH variation occur and thus the overall structure and dynamics of DENV C protein is maintained.

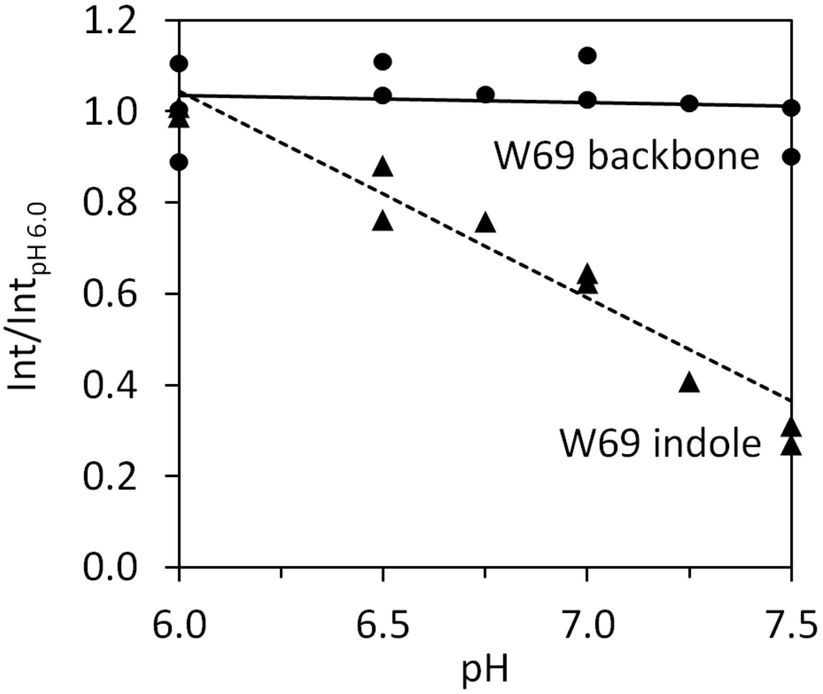

Figure S3. DENV C W69 demonstrates the local probing of this approach. Int/Int$_{pH\ 6.0}$ values of W69 backbone N-H (circles and full line) and indole N-H (triangles and dashed line). Lines are fits of equation 1 to the data. The backbone amide is not affected by pH (Int$_{pH\ 7.5}$/Int$_{pH\ 6.0}$ value of 0.954 ± 0.074) while the indole N-H group value varies significantly (Int$_{pH\ 7.5}$/Int$_{pH\ 6.0}$ is 0.291 ± 0.018).

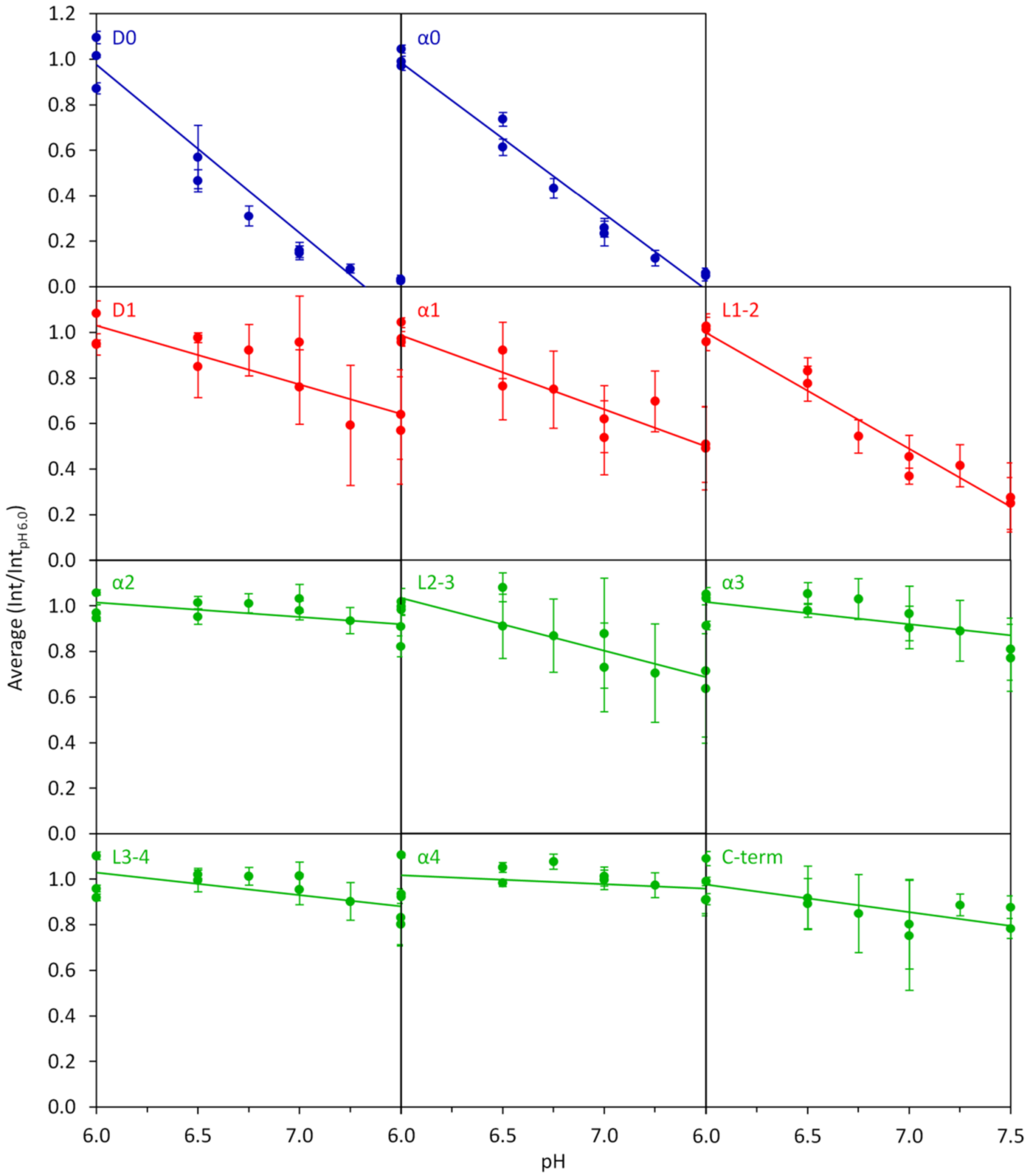

Figure S4. Average intensities ratio of DENV C secondary structure domains as a function of pH. In all graphs, lines are fits of equation 1 to the experimental data, from which slopes were extracted (Fig. 5b). Among the secondary structure domains, α0 and D0 are those that vary the most in intensity with pH, with α1 and L1-2 presenting an intermediate behavior.

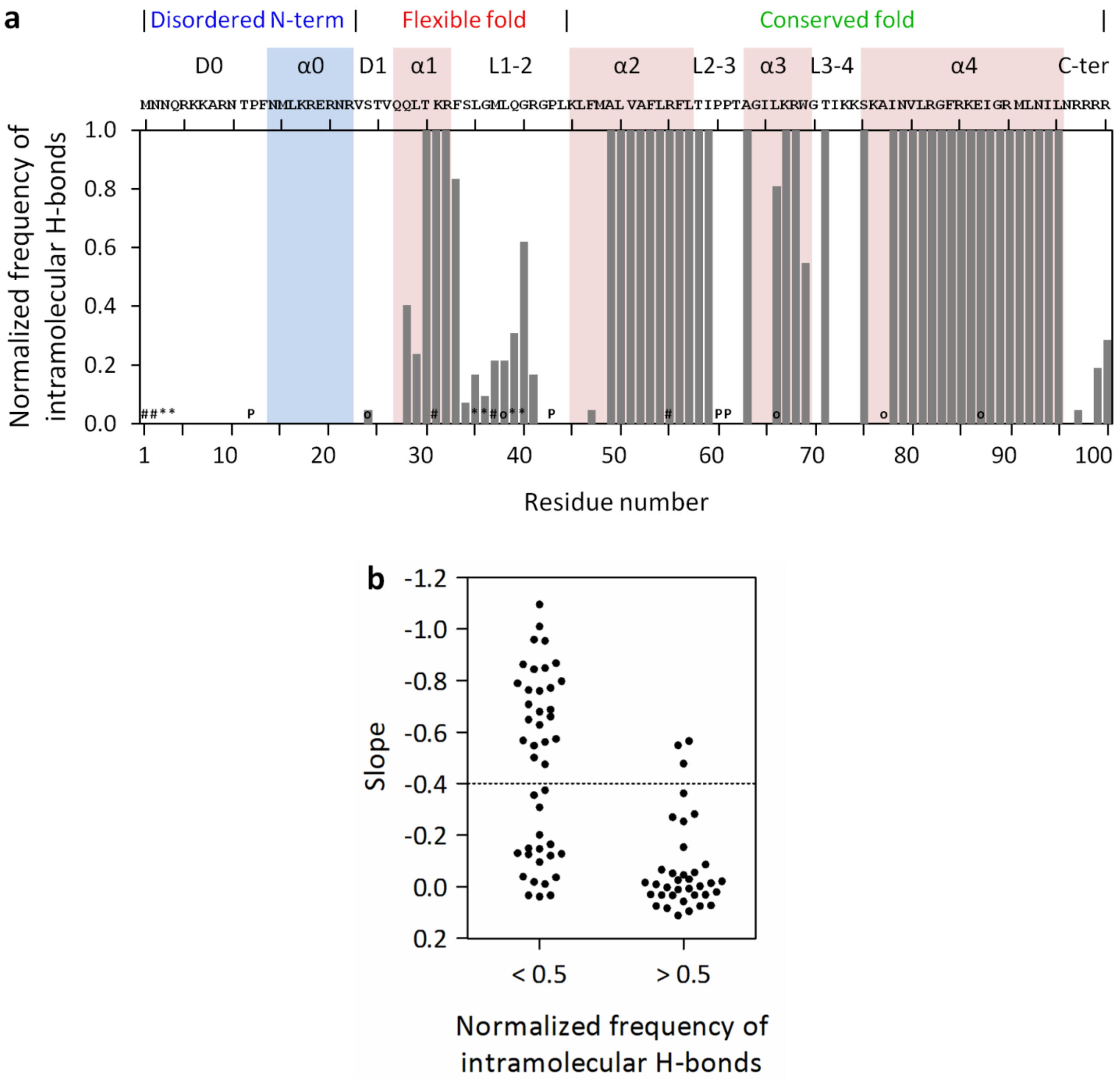

Figure S5. Intramolecular H-bond frequency of DENV C backbone N-H groups and its relationship with the slopes obtained from equation 1. (**a**) Normalized frequency of intramolecular H-bonds involving each DENV C backbone N-H group (based on PDB structure file 1R6R[2]). For details on the protein structural information and symbols, on top and within the graph (respectively), please refer to the legend of Fig. 2. (**b**) Correlation of the slopes information with the normalized frequency of intramolecular H-bonds, showing that the majority of slope values below -0.4 (dots above the line) arises from residues with normalized frequency of intramolecular H-bonds lower than 0.5. Note that the initial residues from α-helices are, in general, not performing H-bonds and, therefore, can exchange hydrogens with the solvent if exposed to it.

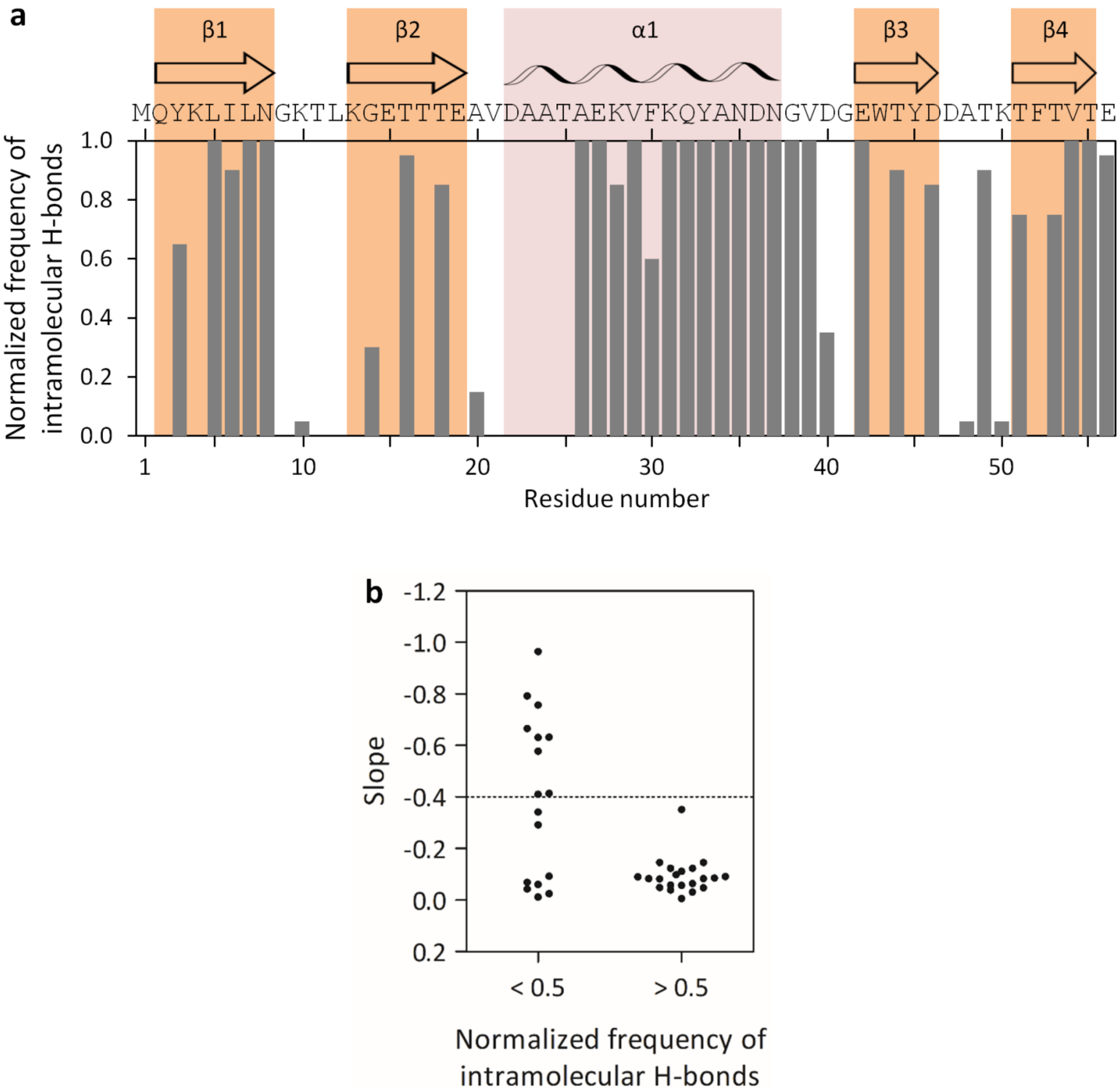

Figure S6. Intramolecular H-bond frequency of GB1 backbone N-H groups and its relationship with the slopes obtained from equation 1. (**a**) Normalized frequency of intramolecular H-bonds involving each GB1 backbone N-H group (based on PDB structure file 5JVX[3]). (**b**) Correlation of the slopes information with the normalized frequency of intramolecular H-bonds, showing that most of the slope values below -0.4 (dots above the line) arise from residues with normalized frequency of intramolecular H-bonds lower than 0.5. As indicated by our analysis (Figs. 7 and 8 of the main manuscript), the β-sheet named β2 is the least protected in terms of intramolecular H-bonds and, thus, the most able to interact with the solvent (as indicated in Fig. 8)

SUPPLEMENTARY NOTE

Slopes in the context of amide N-H hydrogen exchange theory

In typical studies of amide hydrogen exchange, the protein is lyophilized from the conditions in which one wants to perform the measurements and, then, the lyophilizate should be rapidly dissolved in pure $^{2}H_2O$ and the NMR measurements began as fast as possible[4-11]. Proton ^{1}H-^{15}N HSQC spectra are collected as a function of time, usually during minutes to hours, in order to extract the intensity decays from each of the protein's N-H groups[4-11]. In the HSQC spectra, there is a pH dependent saturation of the N-H hydrogens, which results from water exchange. A similar formalism can be used to understand the pH-dependent saturation in a HSQC spectrum. In these studies, the following reactions are considered (Linderstrøm–Lang kinetic model)[4-11]:

$$\mathrm{N-{}^{1}H_{closed}} \underset{k_{cl}}{\overset{k_{op}}{\rightleftarrows}} \mathrm{N-{}^{1}H_{open}} \qquad \text{(R1)}$$

$$\mathrm{N-{}^{1}H_{open}} + {}^{1}\mathrm{H}/{}^{2}\mathrm{HO}^{-*}_{\mathrm{solvent}} \overset{k_{rc}}{\rightarrow} \mathrm{N-{}^{1}H/{}^{2}H^{*}_{open}} + {}^{1}\mathrm{HO}^{-}_{\mathrm{solvent}} \qquad \text{(R2)}$$

In the first reaction scheme, R1, a specific amino acid residue amide group is in equilibrium between solvent inaccessible conformation (N-H_{closed}) and solvent exposed conformation (N-H_{open}). These two conformations exchange through the opening and the closing rate constants, k_{op} and k_{cl}, respectively (these are intrinsic rate constants related with protein structure and dynamics, which depend on the local microenvironment). The closed/open conformational equilibrium contributes to the observed NMR peak intensity for a specific N-H group of an amino acid residue. Only the N-H_{open} conformation exchanges the amide proton with the solvent according to the R2 scheme. k_{rc} is the intrinsic amino acid residue amide hydrogen exchange rate constant. Here, we are not considering acidic catalysis since, at pH values higher than 4.5 (in general), the hydrogen exchange is mainly catalyzed by the $^{1}HO^{-}$ or $^{2}HO^{-}$ ions in solution (represented as $^{1}H/^{2}HO^{-}_{solvent}$ because we used a mixture of 90% $^{1}H_2O$ and 10% $^{2}H_2O$ as solvent)[4-11]. Thus, k_{rc} is proportional to $^{1}H/^{2}HO^{-}$ concentration ([$^{1}H/^{2}HO^{-}$]) and it can be expressed as a function of pH (from now on, denoted $k_{rc,pH}$):

$$k_{rc,pH} \propto [{}^{1}\mathrm{H}/{}^{2}\mathrm{HO}^{-}] \Rightarrow k_{rc,pH} = \alpha \times 10^{pH} \qquad \text{(A1)}$$

where α is a pH independent constant that is specific for each type of amino acid[4-11].

From schemes R1 and R2, the NMR peak intensity decays are described by[4-11]:

$$\mathrm{Int} = \mathrm{Int}_{t=0}\, e^{-\left(\frac{k_{op}\, k_{rc,pH}}{k_{op}+k_{cl}+k_{rc,pH}}\right)t} \qquad \text{(A2)}$$

where t is the saturation time encoded in the HSQC pulse sequence, while Int and $\mathrm{Int}_{t=0}$ are the NMR peak intensities as a function of the saturation time and at time 0, respectively. The theoretical values of $k_{rc,pH}$ can be estimated according to references[4-11]. Thus, considering that a residue is part

of a disordered domain and fully exposed to the solvent (*i.e.*, $k_{op} >> k_{cl}$ and $k_{op} >> k_{rc,pH}$), equation A2 can be simplified to:

$$\mathrm{Int} = \mathrm{Int}_{t=0}\, e^{-k_{rc,pH}\, t} \qquad \text{(A3)}$$

Here, we are varying the pH in a controlled way, while the acquisition time of the measurements and all the HSQC pulses and delays are maintained constant (*t* is the same for every pH), to access to the spectral intensity differences at different pH values. Importantly, we are considering that the protein structure and dynamics behavior does not change significantly in the tested pH range. With this, it is assumed that k_{op} and k_{cl} are independent of pH and, thus, both of these rates are constant for each amino acid. Thus, the normalized NMR peak intensities ($\mathrm{Int}/\mathrm{Int}_{pH\ 6.0}$) for a specific N-H group are described as follows:

$$\frac{\mathrm{Int}}{\mathrm{Int}_{pH\ 6.0}} = \frac{\mathrm{Int}_{t=0,pH}}{\mathrm{Int}_{t=0,pH\ 6.0}} \times \frac{e^{-\left(\frac{k_{op}\, k_{rc,pH}}{k_{op}+k_{cl}+k_{rc,pH}}\right)t}}{e^{-\left(\frac{k_{op}\, k_{rc,pH\ 6.0}}{k_{op}+k_{cl}+k_{rc,pH\ 6.0}}\right)t}} = \underbrace{\left(\mathrm{Int}_{t=0,pH}/\mathrm{Int}_{t=0,pH\ 6.0}\right)}_{\text{Protein dynamics contribution}} \times \underbrace{e^{-\left(\frac{k_{op}\, k_{rc,pH}}{k_{op}+k_{cl}+k_{rc,pH}} - \frac{k_{op}\, k_{rc,pH\ 6.0}}{k_{op}+k_{cl}+k_{rc,pH\ 6.0}}\right)t}}_{\text{Hydrogen exchange contribution}}$$

$$\cong e^{-\left(\frac{k_{op}\, k_{rc,pH}}{k_{op}+k_{cl}+k_{rc,pH}} - \frac{k_{op}\, k_{rc,pH\ 6.0}}{k_{op}+k_{cl}+k_{rc,pH\ 6.0}}\right)t} \qquad \text{(A4)}$$

where $k_{rc,pH}$ and $k_{rc,pH\ 6.0}$ are the specific amino acid random chain amide hydrogen rate constants at a specific pH value and at pH 6.0, respectively. Note that equation A4 has two contributions. The contribution from protein dynamics can be important if there is a significant change in the N-H transverse relaxation or a difference in conformational exchange ($R_2 = R_2^{DD,CSA} + R_{ex}$) caused by the variation in pH, relative to pH 6.0. This would lead to changes in line broadening and, thus, in the NMR peak intensities at *t* = 0. Considering that there is no significant change in R_2, based on the measurements shown in Fig. S2, the protein dynamics contribution reduces to 1, especially in disordered regions where the conformational exchange contribution (R_{ex}) is negligible. Thus, only the hydrogen exchange contribution is relevant to equation A4.

In the case of the disordered residues fully exposed to the solvent $k_{cl} << k_{op}$ (and $k_{cl} << k_{rc,pH\ 6.0}$, the exchange limit EX1[11]) and so $\mathrm{Int}/\mathrm{Int}_{pH\ 6.0}$ should vary simply according to equation A5:

$$\frac{\mathrm{Int}}{\mathrm{Int}_{pH\ 6.0}} \cong e^{-\left(\frac{k_{op}\, k_{rc,pH}}{k_{op}+k_{rc,pH}} - \frac{k_{op}\, k_{rc,pH\ 6.0}}{k_{op}+k_{rc,pH\ 6.0}}\right)t} \qquad \text{(A5)}$$

There are two limits for equation A5; one that is pH independent, where $k_{op} << k_{rc}$ (especially $k_{op} << k_{rc,\ pH\ 6.0}$), which reduces equation A5 to 1 (*i.e.*, very slow solvent exposure, no H exchange):

$$\frac{\mathrm{Int}}{\mathrm{Int}_{pH\ 6.0}} \cong e^{-\left(\frac{k_{op}\, k_{rc,pH}}{k_{rc,pH}} - \frac{k_{op}\, k_{rc,pH\ 6.0}}{k_{rc,pH\ 6.0}}\right)t} = e^{-\left(k_{op}-k_{op}\right)t} = 1 \qquad \text{(A6)}$$

and another that is pH-dependent, where $k_{op} >> k_{rc}$:

$$\frac{\mathrm{Int}}{\mathrm{Int}_{pH\ 6.0}} \cong e^{-\left(\frac{k_{op}\, k_{rc,pH}}{k_{op}} - \frac{k_{op}\, k_{rc,pH\ 6.0}}{k_{op}}\right)t} = e^{-\left(k_{rc,pH}-k_{rc,pH\ 6.0}\right)t} \qquad \text{(A7)}$$

Equation A7 is pH independent, if the folding equilibrium is unchanged within the pH range of study. Note that equation A7 could be derived directly from equation A3, considering the intensity ratio between the two pH values equal to 1. Under the experimental conditions studied in this manuscript, we observed disordered residues within the limit of equation A7. Thus, equations A4 to A7 entirely explain the $Int_{pH\ 7.5}/Int_{pH\ 6.0}$ analysis (Figs. 2a and 2b) and the shape of the trends observed when $Int/Int_{pH\ 6.0}$ is plotted against pH (Fig. 4).

To explain the slopes analysis, since the slope is the average value (within the tested pH range) of the $Int/Int_{pH\ 6.0}$ derivative against pH, we must calculate the derivative of equation A4 against pH. Combining equation A1 with equation A4 and considering that $k_{rc,pH\ 6.0}$ is constant for a specific residue, A4 becomes:

$$\frac{\mathrm{Int}}{\mathrm{Int}_{pH\ 6.0}} = e^{-\left(\frac{k_{op}\ (\alpha\times10^{pH})}{k_{op}+k_{cl}+(\alpha\times10^{pH})} - \frac{k_{op}\ k_{rc,pH\ 6.0}}{k_{op}+k_{cl}+k_{rc,pH\ 6.0}}\right)t} \qquad \text{(A8)}$$

Deriving equation A8 in order to pH yields:

$$\frac{d\left(\frac{\mathrm{Int}}{\mathrm{Int}_{pH\ 6.0}}\right)}{d\ \mathrm{pH}} = -\frac{\ln(10)\ k_{op}\left(k_{op}+k_{cl}\right)(\alpha\times10^{pH})\ t}{\left(k_{op}+k_{cl}+(\alpha\times10^{pH})\right)^2}\times e^{-\left(\frac{k_{op}\ (\alpha\times10^{pH})}{k_{op}+k_{cl}+(\alpha\times10^{pH})} - \frac{k_{op}\ k_{rc,pH\ 6.0}}{k_{op}+k_{cl}+k_{rc,pH\ 6.0}}\right)t}$$

$$= -\frac{2.303\ k_{op}\left(k_{op}+k_{cl}\right)(\alpha\times10^{pH})\ t}{\left(k_{op}+k_{cl}+(\alpha\times10^{pH})\right)^2}\times\frac{\mathrm{Int}}{\mathrm{Int}_{pH\ 6.0}} \qquad \text{(A9)}$$

In which the factor that includes pH can be substituted to $k_{rc,pH}$, via A1 again, becoming:

$$\frac{d\left(\frac{\mathrm{Int}}{\mathrm{Int}_{pH\ 6.0}}\right)}{d\ \mathrm{pH}} = -\frac{2.303\ k_{op}\left(k_{op}+k_{cl}\right)k_{rc,pH}\ t}{\left(k_{op}+k_{cl}+k_{rc,pH}\right)^2}\times\frac{\mathrm{Int}}{\mathrm{Int}_{pH\ 6.0}} \qquad \text{(A10)}$$

Analogously, in the limit for the disordered fully exposed residues ($k_{op} >> k_{cl}$ and $k_{op} >> k_{rc,pH}$; described by A7), the derivative in order to pH becomes a simpler version of A10:

$$\frac{d\left(\frac{\mathrm{Int}}{\mathrm{Int}_{pH\ 6.0}}\right)}{d\ \mathrm{pH}} = -2.303\ k_{rc,pH}\ t\times\frac{\mathrm{Int}}{\mathrm{Int}_{pH\ 6.0}} \qquad \text{(A11)}$$

The values of $k_{rc,pH}$ can be estimated for a given pH value at a fixed temperature[4-11]. As such, at constant time (equal for all the spectra acquired), equation A11 gives a theoretical limit for the slope of every amino acid residue. Moreover, the comparison between experimentally measured and theoretical slopes can retrieve direct information about the dynamics of the N-H groups of a protein. If the experimental slope is statistically equal to the theoretical slope, which was calculated assuming that the residue is disordered, one can assume that the residue is, in fact, intrinsically disordered and exposed to the water. Very stable residues have experimental slopes of 0, meaning that $k_{cl} >> k_{op}$ and $k_{cl} >> k_{rc,pH}$. As a corollary of this, for residues whose slope is between 0 and the theoretical slope, one can establish that k_{op} and/or k_{cl} are playing an important role in the observed

behavior. Thus, that specific N-H group is transiently exposed to the solvent and, possibly, in conformational exchange.

In summary, we explain the N-H groups behavior observed in this study in light of the current knowledge on the NMR hydrogen exchange phenomenon. From both analysis, the $Int_{pH\ 7.5}/Int_{pH\ 6.0}$ (Figs. 2a and 2b) and the slopes (Fig. 5a), it is difficult to reliably extract the conformational exchange rates k_{op} and k_{cl}, since they are mutually influenced and also influenced by $k_{rc,pH}$ in the equations. Thus, the simpler analysis presented by this work is easier to apply to a protein structure/dynamics study in order to quickly evaluate the N-H groups solvent accessibility.

SUPPLEMENTARY METHODS

DENV C – heterologous expression and purification

DENV C protein purification protocol was optimized from previous studies[2,12-16]. The protein was expressed in *E. coli* BL21-CodonPlus transformed with a pET-21a plasmid with a gene encoding the capsid protein of DENV serotype 2, strain New Guinea C (NCBI ID AAC59275, corresponding to amino acids 1-100 of the polyprotein)[14-17]. After transformation, cells were grown on LB-agar plates with 100 µg/mL ampicillin and 34 µg/mL chloramphenicol, at 37 ºC. One *E. coli* colony of the overnight plate culture was transferred to a freshly prepared 5 mL LB medium with 100 µg/mL ampicillin and 34 µg/mL chloramphenicol and, after ~8 h incubation at 200 rpm and 37 ºC, that culture medium was added to 30 mL of M9 minimal medium containing 1 g/L of [^{15}N]-NH_4Cl, 5 g/L of glucose, 100 µg/mL ampicillin and 34 µg/mL chloramphenicol. After overnight incubation at 200 rpm and 37 ºC, the 30 mL culture was used to inoculate 0.97 L of the supplemented M9 minimal medium indicated above (to obtain ^{15}N-uniformelly labeled protein), and grown for ~5 h at 200 rpm and 37 ºC. Protein expression was induced with 0.5 mM isopropyl β-D-1-thiogalactopyranoside (IPTG) when OD_{600} = 0.9 ± 0.1, and culture stayed ON at 20 ºC, 200 rpm.

After heterologous protein expression, the cell culture was centrifuged at 12,000 *g* for 20 min at 4 ºC, and the supernatant was discarded. The pellet was resuspended in about 70 mL of Buffer A (25 mM HEPES, pH 7.4, 0.2 M NaCl, 1 mM EDTA, 5% (v/v) glycerol and 10 µM protease inhibitor mix, including phenylmethylsulfonyl fluoride (PMSF), pepstatin, leupeptin, E64 and bestatin). Cells were lysed by freezing and thawing (with liquid N_2 and 42 ºC), totalizing 10 to 20 cycles, followed by 10 to 15 cycles of sonication (with the bottle containing the lysate on ice). Crystalline NaCl was added to the cell lysate to achieve a final NaCl concentration of 2 M to dissociate the DENV C bound to DNA or RNA. The lysate was left on agitation for 1 h, at 4 ºC. Precipitation was achieved by the addition of $(NH_4)_2SO_4$ at 30% of saturation and agitation for 1 h on ice. Then, the lysate was centrifuged at 17,000 *g* for 30 min at 4 ºC and more $(NH_4)_2SO_4$ was added to the supernatant to make 60% of the saturation. The suspension was again left on agitation for 1 h on ice and centrifuged at 17,000 *g* for 30 min at 4 ºC. The pellet was resuspended with Buffer A and centrifuged at 30,000 *g* for 15 min at 4 ºC. Supernatants and pellets of all centrifugation steps were analyzed through 18% SDS-PAGE for quality and yield check. The final supernatant was injected onto a HiTrap heparin column of 5 mL (previously washed and equilibrated with Buffer A), coupled to a peristaltic pump P-1, at a 5 mL/min flow rate. Steps of Buffer A with increasing NaCl concentration (100 mL of 1.0 M, 50 mL of 1.5 M and 100 mL of 2 M) were employed and DENV C eluted at 1.5 M NaCl. The 5 mL fractions containing DENV C protein were confirmed by 18% SDS-PAGE and absorbance at 280 nm to infer the protein purity

and concentration. They were then pooled and dialyzed with 3.5 kDa cut-off membrane tube against buffer containing 55 mM KH_2PO_4, pH 6.0, 550 mM KCl. DENV C was concentrated with Amicon Ultra-4 Centrifugal Filter of 10 kDa cut-off and then stored at -20 ºC.

GB1 – heterologous expression and purification

The pET11a plasmid containing the gene encoding T2Q B1 immunoglobulin G binding domain of streptococcal protein G (GB1) was kindly provided by Professor Gary Pielak, from University of North Carolina at Chapel Hill. The T2Q mutation prevents N-terminal deamidation. This form is mentioned here as wild type (WT) or only "GB1". The isolation and purification of ^{15}N,^{13}C enriched GB1 was optimized from previous studies[18-21]. The protein was expressed in the BL21 (DE3) *Escherichia coli* strain. The cells transformed with the GB1 expression plasmid were grown with shaking at 37 ºC and 180 rpm, in M9 minimal medium enriched with [^{15}N]-NH_4Cl (2.5 g/L) and [^{13}C]-glucose (4 g/L). Expression was induced by 1 mM IPTG when an OD_{600nm} of 0.6 was reached. After 3 h induction in the same conditions, the cells were harvested for 12 mins at 6,400 *g* and frozen at -20ºC overnight. The cell pellet was resuspended into lysis buffer (10 mM Tris-HCl, 1 mM EDTA, pH 7.5) preheated to 80ºC. The sample was stirred and heated until the temperature reached 80 °C for 5 min. The lysed cells were cooled on ice for 10 min and then centrifuged at 29,000 *g* for 30 min. The supernatant was dialyzed overnight against 20 mM Tris-HCl, pH 7.5. The dialyzed supernatant was purified via anion exchange chromatography with a HiTrap Q HP, using diethylaminoethyl cellulose resin, on an AKTA start chromatograph. Buffer A (20 mM Tris·HCl, pH 7.5) was used to load the crude lysate onto the column and elute impurities. Buffer B (20 mM Tris-HCl, 1 M NaCl, pH 7.5) was used to produce a linear gradient of 0–400 mM NaCl. Fractions containing GB1 were pooled and concentrated with Amicon Centricons with 3-kDa molecular-mass centrifugal membranes by centrifuging at 4000 *g*, at 4ºC, for further purification by size exclusion chromatography (Superdex 75 10/300 GL column) with a running buffer of 20 mM potassium phosphate, 50 mM NaCl, pH 6.0. The pure fractions were pooled, extensively dialyzed against Milli-Q water, frozen, and lyophilized. The final concentration of the protein was kept around 1.5 mg/mL and the yields obtained were around 30 mg of protein *per* liter of cell culture.

DENV C – NMR spectroscopic analysis of the pH variation

NMR experiments were performed at 298.15 K in a Bruker Avance III 800 MHz equipped with a triple resonance (^{1}H, ^{13}C, ^{15}N) probe. Gradient selection ^{1}H-^{15}N HSQC spectra with sensitivity enhancement (namely, hsqcetf3gpsi from Bruker) were acquired at 800.4 MHz with 1024 × 512 complex points (echo-anti-echo) with 16 accumulations for the first spectrum at pH 6.0 (~2 h), 64 accumulations at the first spectrum at pH 7.5 (~8 h) and 8 accumulations for all the other spectra

(~1 h). Quadrature detection in indirect dimension was done using States-TPPI (time proportional phase incrementation) for ^{1}H and echo-anti-echo for ^{15}N. DENV C chemical shifts were referenced with respect to the ^1H_2O signal at 4.77 p.p.m. (pH 6.8, 25 ºC) relative to DSS (4,4-dimethyl-4-silapentane-1-sulfonic acid). NMR spectra were processed using NMRPipe[22] and analyzed with NMRViewJ[23]. Chemical shift assignments were obtained by direct comparison with the deposited data for DENV C (Biomagnetic Resonance Data Bank (BMRB) ID 5973[2]) and with the peak lists from[15], taking into account NMR spectra acquired at different pH values. With this approach, 85 % of the backbone amidic resonances were unambiguously assigned. From the unassigned residues: M1, N2, K31, M37 and R55 were not assigned in the previously determined NMR structure[2] (marked with '#'); residues P12, P43, P60 and P61 are prolines and do not appear in the ^{1}H-^{15}N HSQC spectrum (marked with 'P'); and residues N3, Q4, L35, G36, Q39 and G40, which were previously assigned[2], were not found in the spectrum at pH 6.0, probably due to line broadening (marked with '*'). Residues S24, L38, L66, A77 and E87 were found, but were impossible to analyze due to the overlapping with other residues (marked with 'o'). The remaining 80 residues were thus analyzable for their peak variation with pH.

The pulse sequence employed here is a Bruker standard version of a gradient-enhanced HSQC sequence in which coherence selection is achieved by means of pulsed field gradient (hsqcetf3gpsi). As HSQC peak intensities are influenced by hydrogen exchange with the water (which is the subject of this work), the labile amide hydrogens are affected. However, this effect is constant if measurements are performed in identical conditions (as it is the case here) and, since we use a semi-empirical approach, this has no substantial influence on both data treatment and conclusions obtained via the described method.

DENV C – NMR spectroscopic analysis of the pH variation

NMR experiments were also performed at 298.15 K in a Bruker Avance III 800 MHz equipped with a triple resonance (^{1}H, ^{13}C, ^{15}N) probe. Spectra were processed using NMRPipe[22] and analyzed with NMRViewJ[23]. Spectra for ^{15}N R_2 determination were acquired as pseuso-3D, with 2D ^{1}H-detected, ^{15}N-edited HSQC experiments, implementing standard pulse sequences[24-27]. Spectra were recorded with spectral widths of 1024 × 256 complex points in the ^{1}H and ^{15}N dimensions, respectively. The field strength of the CPMG refocusing train was 500 Hz and a 1.2 ms delay was used between the refocusing pulses[28,29]. The effects of cross relaxation between ^{1}H-^{15}N dipolar and ^{15}N chemical shift anisotropy were removed by applying ^{1}H 180° pulses during relaxation delays[30]. The inter-scan delay for the measurements was 2 s. ^{15}N R_2 relaxation rates were measured from spectra with different relaxation delays: 0.016, 0.048, 0.080 (triplicate), 0.112, 0.144, 0.176, 0.224 and 0.288 s.

Single exponential decays were fitted to the data on GraphPad Prism v5 software using the non-linear least squares method and SE values were obtained from the error in R_2 calculation. Regarding R_2 values (Fig. S2), despite the loss of information from residues K6 to N15, L17, N21, T25, G42, L44, I59, T62 and R99 (which are not detectable at pH 7.5), and the gain of information from L29, A52, L66, K67, E87, I88 and M91 (which, at pH 6.0, were superimposed with residues that disappeared at pH 7.5, precluding the calculation of R_2 at pH 6.0), the majority of residues that could be studied at pH 6.0 (70 residues) were also found at pH 7.5 (53 out of 60). Despite residues V80 to G83 and R90 to L95, located in α4 helix, increase their R_2 values by approximately 8.1 s^{-1}, the majority of R_2 values of DENV C were constant between pH 6.0 and 7.5 (consult also Fig. S2).

GB1 – NMR spectroscopic analysis of the pH variation

NMR experiments were performed at 303.15 K in a Bruker Avance II+ 600 MHz spectrometer equipped with 5-mm TCI cryoprobe. ^1H-^{15}N HSQC spectra were acquired at 600.13 MHz with 2048 × 128 complex points, with 2 accumulations for GB1 buffer solutions at pH 6.5, 7.3, 7.6 and 8.0. Proton chemical shifts were referenced against internal DSS, while nitrogen chemical shifts were referenced indirectly to DSS using the absolute frequency ratio. Data were processed using Bruker TopSpin™ 4.0 and analyzed with CCPNMR[31] for cross-peak assignment and height extraction. GB1 assignments are based on Gronenbron *et al.*[21].

Amino acid residues pK_a assessment

The DENV C theoretical isoelectric point was calculated in ProtParam web server (http://web.expasy.org/protparam/). The average pK_a and standard deviation (SD) for the titratable protein amino acid residues side-chains, as well as for the N- and C-termini, were directly obtained from the literature on Protein pK_a Database (PPD v1.0)[1], which is a database of experimentally determined pK_a values from 163 proteins (http://www.ddg-pharmfac.net/ppd/PPD/pKahomepage.htm)[1]. There is a low availability of pK_a values for arginine (R) residues[1], due to the high pH that must be reached to determine them. As there is no SD available for this amino acid, on Fig. S1 we choose to plot the SD for R as the average of SD values of the other amino acids.

Protein structure visualization

UCSF Chimera v1.9 software[32] was used to prepare the protein structure figures. PBD file 1R6R[2], conformer 21 (the representative average of the lowest energy conformers calculated) was used as experimentally determined DENV C structure (lacking the first 20 residues). A molecular dynamics energy minimized model containing predicted DENV C N-terminal structure combined with the experimental structure[33], collected at the 20 ns time frame, was employed to display the possible

alternative configuration for the entire homodimer (Fig. 1b). A similar approach was followed for GB1 studies (based on PDB structure file 5JVX[3]).

Calculation of the normalized frequency of intramolecular H-bonds

The normalized frequency of intramolecular H-bonds was calculated from the known DENV C protein structure (PDB ID: 1R6R[2]). Briefly, we extracted the frequency of each backbone amide group H-bond via UCSF Chimera v1.9 software[32] (using standard distance values to define H-bonding) for the 42 DENV C monomer models contained in the 1R6R structure file. Then, the frequencies obtained were divided by 42 to obtain the normalized frequency. Few backbone N-H groups were establishing more than one H-bond and, when divided by 42, the value was greater than 1. Thus, all values greater than 1 were assumed to have normalized frequency equal to 1 (100% in H-bond). As the first 20 residues are not present in the structure file, due to their intrinsically disordered nature when the protein is free in solution (which was the case in this study), an H-bond frequency of zero was assumed for those residues. The statistical analysis performed is described below. A similar approach was followed to study GB1 (PDB file 5JVX[3]).

Statistical analysis

Regarding data acquisition, different numbers of spectra were collected for the several pH values tested with DENV C: pH 6.0, n = 3; pH 6.5, n = 2; pH 6.75, n = 1; pH 7.0, n = 2; pH 7.25, n = 1; pH 7.5, n = 2. Thus, for the calculation of the $Int_{pH\ 7.5}/Int_{pH\ 6.0}$, $n_{total} = 5$, and for the calculation of slopes, the $n_{total} = 11$. For the average values, propagated standard error (SE) was calculated as the standard deviation (SD) divided by the square root of the number of points used for the calculation. The SE of $Int_{pH\ 7.5}/\ Int_{pH\ 6.0}$ was calculated according to equation 2:

$$SE_{Ratio} = \left|\frac{Int_{pH\ 7.5}}{Int_{pH\ 6.0}}\right| \times \sqrt{\left(\frac{SE_{pH\ 7.5}}{Int_{pH\ 7.5}}\right)^2 + \left(\frac{SE_{pH\ 6.0}}{Int_{pH\ 6.0}}\right)^2} \qquad (2)$$

For the slopes, SE was obtained directly from the error in the calculation of slopes through the fitting of equation 1 to the data, performed on GraphPad Prism 5 software using the least squares method. The significance of the difference between slopes from residues with normalized frequency of intramolecular H-bonds below 0.5 and slopes from residues with normalized frequency of intramolecular H-bonds above 0.5 was also analyzed using GraphPad Prism 5 software, by employing a two-tailed unpaired *t*-test with Welch's correction (not assuming equal variances).

SUPPLEMENTARY INFORMATION REFERENCES

1 Toseland, C. P., McSparron, H., Davies, M. N. & Flower, D. R. PPD v1.0 – an integrated, web-accessible database of experimentally determined protein pKa values. *Nucleic Acids Res.* 34, D199-203 (2006).

2 Ma, L., Jones, C. T., Groesch, T. D., Kuhn, R. J. & Post, C. B. Solution structure of dengue virus capsid protein reveals another fold. *Proc. Natl. Acad. Sci. U.S.A.* 101, 3414-3419 (2004).

3 Andreas, L. B. *et al.* Structure of fully protonated proteins by proton-detected magic-angle spinning NMR. *Proc. Natl. Acad. Sci. U.S.A.* 113, 9187-9192(2016).

4 Englander, S. W. & Mayne, L. Protein folding studied using hydrogen-exchange labeling and two-dimensional NMR. *Annu. Rev. Bioph. Biomol. Struct.* 21, 243-265(1992).

5 Bai, Y., Milne, J. S., Mayne, L. & Englander, S. W. Primary structure effects on peptide group hydrogen exchange. *Proteins* 17, 75-86 (1993).

6 Connelly, G. P., Bai, Y., Jeng, M. F. & Englander, S. W. Isotope effects in peptide group hydrogen exchange. *Proteins* 17, 87-92 (1993).

7 Bai, Y., Sosnick, T. R., Mayne, L. & Englander, S. W. Protein folding intermediates: native-state hydrogen exchange. *Science* 269, 192-197 (1995).

8 Koide, S., Jahnke, W. & Wright, P. E. Measurement of intrinsic exchange rates of amide protons in a ^{15}N-labeled peptide. *J. Biomol. NMR* 6, 306-312 (1995).

9 Englander, S. W., Mayne, L., Bai, Y. & Sosnick, T. R. Hydrogen exchange: the modern legacy of Linderstrom-Lang. *Protein Sci*. 6, 1101-1109(1997).

10 Hwang, T. L., van Zijl, P. C. & Mori, S. Accurate quantitation of water-amide proton exchange rates using the phase-modulated CLEAN chemical EXchange (CLEANEX-PM) approach with a Fast-HSQC (FHSQC) detection scheme. *J. Biomol. NMR* 11, 221-226 (1998).

11 Krishna, M. M., Hoang, L., Lin, Y. & Englander, S. W. Hydrogen exchange methods to study protein folding. *Methods* 34, 51-64 (2004).

12 Jones, C. T. *et al.* Flavivirus capsid is a dimeric alpha-helical protein. *J. Virol.* 77, 7143-7149 (2003).

13 Samsa, M. M. *et al.* Dengue virus capsid protein usurps lipid droplets for viral particle formation. *PLoS Pathog.* 5, e1000632 (2009).

14 Carvalho, F. A. *et al.* Dengue virus capsid protein binding to hepatic lipid droplets (LD) is potassium ion dependent and mediated by LD surface proteins. *J. Virol.* 86, 2096-2108 (2012).

15 Martins, I. C. *et al.* The disordered N-terminal region of dengue virus capsid protein contains a lipid-droplet-binding motif. *Biochem. J.* 444, 405-415 (2012).

16 Faustino, A. F. *et al.* Dengue virus capsid protein interacts specifically with very low-density lipoproteins. *Nanomedicine: NBM* 10, 247-255 (2014).

17 Irie, K., Mohan, P. M., Sasaguri, Y., Putnak, R. & Padmanabhan, R. Sequence analysis of cloned dengue virus type 2 genome (New Guinea-C strain). *Gene* 75, 197-211 (1989).

18 Lindman, S. *et al.* Salting the charged surface: pH and salt dependence of protein G B1 stability. *Biophys. J.* 90, 2911-2921 (2006).

19 Monteith, W. B. & Pielak, G. J. Residue level quantification of protein stability in living cells. *Proc. Natl. Acad. Sci. U.S.A.* 111, 11335-11340 (2014).

20 Gallagher, T., Alexander, P., Bryan, P. & Gilliland, G. L. Two crystal structures of the B1 immunoglobulin-binding domain of streptococcal protein G and comparison with NMR. *Biochemistry* 33, 4721-4729 (1994).

21 Gronenborn, A. M. *et al.* A novel, highly stable fold of the immunoglobulin binding domain of streptococcal protein G. *Science* 253, 657-661 (1991).

22 Delaglio, F. *et al.* NMRpipe - a multidimensional spectral processing system based on Unix Pipes. *J. Biomol. NMR* 6, 277-293 (1995).

23 Johnson, B. A. & Blevins, R. A. NMR View: A computer program for the visualization and analysis of NMR data. *J. Biomol. NMR* 4, 603-614(1994).

24 de Medeiros, L. N. *et al.* Backbone dynamics of the antifungal Psd1 pea defensin and its correlation with membrane interaction by NMR spectroscopy. *Biochim. Biophys. Acta* 1798, 105-113 (2010).

25 de Paula, V. S. *et al.* Structural basis for the interaction of human beta-defensin 6 and its putative chemokine receptor CCR2 and breast cancer microvesicles. *J. Mol. Biol.* 425, 4479-4495 (2013).

26 de Paula, V. S., Razzera, G., Barreto-Bergter, E., Almeida, F. C. & Valente, A. P. Portrayal of complex dynamic properties of sugarcane defensin 5 by NMR: multiple motions associated with membrane interaction. *Structure* 19, 26-36 (2011).

27 Farrow, N. A. *et al.* Backbone dynamics of a free and phosphopeptide-complexed Src homology 2 domain studied by ^{15}N NMR relaxation. *Biochemistry* 33, 5984-6003 (1994).

28 Carr, H. Y. & Purcell, E. M. Effects of diffusion on free precession in nuclear magnetic resonance experiments. *Phys. Rev.* 94, 630-638 (1954).

29 Meiboom, S. & Gill, D. Modified spin-echo method for measuring nuclear relaxation times. *Rev. Sci. Instrum.* 29, 688-691 (1958).

30 Palmer, A. G., Williams, J. & McDermott, A. Nuclear magnetic resonence studies of biopolymer dynamics. *J. Phys. Chem.* 100, 13293-13310 (1996).

31 Vranken, W. F. *et al.* The CCPN data model for NMR spectroscopy: development of a software pipeline. *Proteins* 59, 687-696 (2005).

32 Pettersen, E. F. *et al.* UCSF Chimera – a visualization system for exploratory research and analysis. *J. Comput. Chem.* 25, 1605-1612 (2004).

33 Faustino, A. F. *et al.* Understanding dengue virus capsid protein disordered N-Terminus and pep14-23-based inhibition. *ACS Chem. Biol.* 10, 517-526 (2015).
